# Metal Ion-Mediated Regulation of Cell Fate: A Novel Strategy for Synergy with Radiotherapy and Immunotherapy

**DOI:** 10.3390/cancers18050796

**Published:** 2026-02-28

**Authors:** Hanye Xu, Xilin Wang, Hongyi Wang, Runjia Hua, Sihan Chen, Jingwei Xu, Xiaju Cheng

**Affiliations:** 1 Key Laboratory of Radiation Medicine and Protection, School for Radiological and Interdisciplinary Sciences (RAD-X), Collaborative Innovation Center of Radiation Medicine of Jiangsu Higher Education Institutions, The Third Affiliated Hospital of Soochow University, Soochow University, Suzhou 215123, China; 2230509015@stu.suda.edu.cn (H.X.); 2230507008@stu.suda.edu.cn (X.W.); 2330509003@stu.suda.edu.cn (H.W.); 2230509080@stu.suda.edu.cn (R.H.); 2530509040@stu.suda.edu.cn (S.C.); 2 Department of Thoracic Surgery, Suzhou Municipal Hospital Institution, Suzhou 215000, China

**Keywords:** metal ions, cell fate, oxidative stress, metal ion-based immunotherapy, radiotherapy

## Abstract

Metal ions are indispensable for living organisms, participating in essential physiological processes. However, their dysregulated accumulation can trigger cell death and metal overload. The recent discovery of novel regulated cell death modalities, such as cuproptosis and ferroptosis, has significantly advanced the understanding of metal ions in cell fate and immune regulation. This review systematically elucidates the molecular mechanisms underlying metal ion-induced cell death, encompassing oxidative stress, mitochondrial dysfunction, DNA damage, and epigenetic modifications. It further classifies and discusses the hallmarks of various programmed and non-programmed cell death pathways, emphasizing the pivotal role of metal ions in anti-tumor immunity. Moreover, we highlight an emerging therapeutic approach-metal ion-based immunotherapy, which represents a compelling strategy when combined with radiotherapy to amplify therapeutic outcomes. This combined modality holds significant promise for overcoming radiotherapy resistance and expanding its abscopal effects. Finally, current challenges and future research directions in the field of metal immunity are outlined to facilitate the translational application of metal ions in disease therapy and immune modulation.

## Simple Summary

Radiotherapy and immunotherapy are key cancer treatments, yet both face limitations. Metal ions, beyond their traditional roles, are now recognized as important regulators of cell death and immune responses. This review explains how metal ions influence cell fate and anti-tumor immunity, and explores their potential to enhance radiotherapy by boosting immune effects and overcoming resistance. By connecting these mechanisms, our work provides a clear framework for developing new combined therapies, offering valuable insights for researchers and clinicians in oncology, immunology, and related fields.

## 1. Introduction

Metal ions are indispensable co-factors in living organisms, crucial for fundamental biological processes such as enzymatic catalysis, signal transduction, and maintenance of genomic stability [1]. Since their homeostasis is tightly regulated, disruption of their balance leads to oxidative stress [2], mitochondrial dysfunction [3], DNA damage [4], and epigenetic alterations [5], culminating in diverse forms of cell death. While metal ions are historically linked to passive necrosis through oxidative damage, recent research has revolutionized our understanding by revealing that specific metal ions can actively trigger distinct, regulated cell death pathways. For instance, the discovery of ferroptosis [6] and cuproptosis [7] has highlighted the sophisticated and metal-specific nature of these processes. Unlike classical apoptosis, these pathways exhibit unique morphological and biochemical hallmarks, expanding the theoretical paradigm of cell death and presenting novel vulnerabilities for therapeutic intervention, particularly in oncology. Critically, metal ion-driven cell death is not an isolated terminal event but a potent modulator of the immune landscape [8]. It can release damage-associated molecular patterns (DAMPs), activating innate immune sensors like the cyclic GMP-AMP synthase (cGAS)-stimulator of interferon genes (STING) pathway [9] and NOD-like receptor thermal protein domain-associated protein 3 (NLRP3) inflammasome [10]. This bridges cellular stress to adaptive anti-tumor immunity. Furthermore, the metabolism and function of key immune cells, including T cells and tumor-associated macrophages (TAMs), are intrinsically regulated by metal ion availability within the tumor microenvironment (TME) [11]. These insights have propelled the emergence of “metal immunotherapy” as a promising frontier, exploring metals not only as cytotoxic agents but also as vaccine adjuvants [12], immune-metabolic regulators [13], and modulators of the gut microbiome [14].

**Figure 1:**
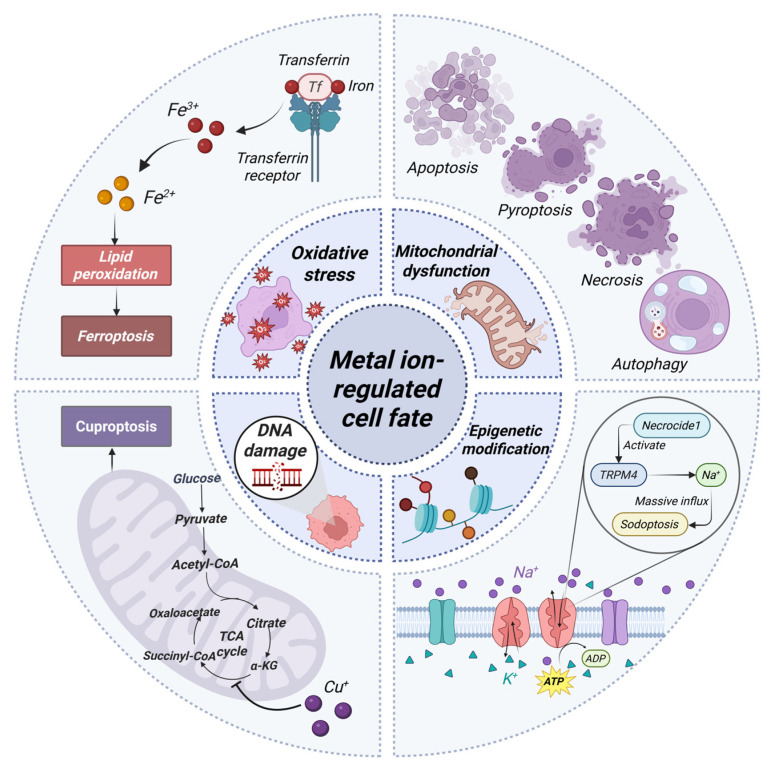
The mechanism and main types of metal ion-regulated cell fate. Created in BioRender. Shen, J. (2026) https://BioRender.com/vl21cit.

It is noteworthy that beyond chemical agents like metal ions, physical modalities play a profound role in driving tumor cell death and reshaping anti-tumor immunity, such as radiotherapy. Radiotherapy exerts direct cytotoxic effects by inducing DNA double-strand breaks (DSBs) [15], generating reactive oxygen species (ROS) [16], and disrupting mitochondrial integrity [17]. More importantly, it can trigger immunogenic cell death (ICD), releasing DAMPs and activating innate immune sensors (e.g., cGAS-STING pathway), thereby converting a localized treatment into a systemic immune response [17]. The strategic enhancement of ICD through nanomedicine-based approaches has emerged as a promising avenue to potentiate the immunogenicity of radiotherapy and overcome the immunosuppressive TME [18]. In recent years, combining radiotherapy with metal-based immunotherapy has emerged as a compelling strategy to amplify therapeutic outcomes. This synergitic effect is achieved through radiosensitizing metal nanomaterials, reprogramming the immunosuppressive TME, or co-administering immune checkpoint inhibitors, which holds significant promise for overcoming radiotherapy resistance and expanding its abscopal effects. Given this rapidly evolving landscape, a comprehensive synthesis is urgently needed. This review aims to systematically delineate the molecular mechanisms underlying metal ion-induced cell death, to classify its various forms, and to critically examine its intricate crosstalk with the immune system. We place special emphasis on the translational potential of these interactions in anti-tumor immunity, culminating in a discussion of metal-based radioimmunotherapeutic strategies (Figure 1). Finally, we will address current challenges and future directions, aiming to provide a cohesive framework that informs both basic research and clinical application in metal treatment.

## 2. Mechanism of Ion-Induced Cell Death

Metal ion-induced cell death represents a sophisticated paradigm in cellular pathology, where a network of molecular mechanisms, including oxidative stress, mitochondrial dysfunction, DNA damage, and genomic instability. These mechanisms do not operate in isolation but engage in dynamic crosstalk and potentiation. This intricate interplay suggests that the pathological impact of metal dyshomeostasis is greater than the sum of its individual pathways, challenging traditional, linear models of toxicity. Therefore, disentangling this interconnected web is critical for the systemic understanding of the intrinsic mechanism, providing the essential foundation for the rational design of targeted therapies. Precision approaches intervene at specific nodes within this network, offering promising strategies against cancers and other diseases where metal ions are central to pathogenesis.

### 2.1. Oxidative Stress

Oxidative stress serves as a central mechanistic hub and signaling nexus through which diverse metal ions exert their cytotoxic effects, integrating metal toxicity into broader cell fate decisions. Multiple heavy metal ions, including cadmium (Cd), lead (Pb), mercury (Hg), and arsenic (As), disrupt redox homeostasis by driving the burst accumulation of ROS, while concurrently depleting endogenous antioxidant defenses such as glutathione (GSH) [19,20] (Table 1). Cd, a prototypical toxicant, and its compounds (e.g., CdCl_2_ and CdSO_4_) induce dose-dependent oxidative damage in neuronal cells [21]. This exposure significantly elevates intracellular ROS and activates key oxidative stress response pathways, including the nuclear factor erythroid 2-related factor 2 (Nrf2)/heme oxygenase 1 (Hmox1)/glutathione S-transferase alpha 3 (Gsta3) axis [21]. While Nrf2 is a master transcription factor that orchestrates the expression of cytoprotective genes and represents an adaptive cellular response [22], chronic Cd exposure can ultimately lead to the exhaustion of this defense system. Furthermore, heavy metals can directly inhibit or deplete crucial antioxidant enzymes like superoxide dismutase (SOD), exacerbating oxidative injury [23]. Different metal ions leverage their distinct chemical properties to channel oxidative stress into specific death signals. For instance, iron (Fe) ions potently catalyze ROS generation via the Fenton reaction, serving as a core driver of ferroptosis. In contrast, cytoplasmic copper (Cu) overload, which leads to cuproptosis via aggregated lipoylated proteins, can also trigger a violent ROS burst through valence cycling (Cu^+^ oxidation), thereby cross-activating multiple pathways such as ferroptosis, autophagy, and apoptosis [2,23,24]. Similarly, excessive zinc (Zn) ions can simultaneously engage mitochondrial, lysosomal, and nuclear pathways, promoting the release of autophagy- and death-related cytokines that culminate in cell death [2,24]. Critically, these metal ions do not act in isolation in vivo but operate within a complex network of synergistic and antagonistic interactions that finely tune the outcome of oxidative stress. For example, Cu has been demonstrated to inhibit ferroptosis [24], whereas Cd, As, and Hg ions can cooperatively promote ferroptosis by increasing intracellular Fe content and suppressing the expression of the key antioxidant enzyme glutathione peroxidase 4 (GPX4) [19].

Beyond the intricate interplay between different metal ions, it is essential to recognize that oxidative stress serves as a convergent mechanism activated by diverse physical and chemical stimuli, including ionizing radiation. Radiotherapy induces a rapid burst of ROS primarily through the radiolysis of water, leading to widespread oxidative damage to cellular components such as lipids. Notably, studies have shown that radiation-generated hydroxyl radicals (•OH) can penetrate lipid bilayers and directly initiate lipid peroxidation chains, thereby potentiating ferroptosis [16]. This overlap in oxidative injury mechanisms reveals that disparate insults (chemical metal ions and physical radiation) can channel into shared downstream pathways, notably oxidative stress, to dictate cell fate. It establishes a novel theoretical framework for designing combination therapies that intentionally co-activate oxidative death pathways, such as using metal-based nanomaterials to radiosensitize tumors or to sustain immunogenic signaling post-radiation. Ultimately, elucidating these shared oxidative circuits informs more effective and synergistic therapeutic regimens against cancer and other oxidative stress-associated diseases.

### 2.2. Mitochondrial Dysfunction

Mitochondrial dysfunction represents a pivotal and integrative node in the spectrum of metal ion-induced cytotoxicity. As the central hub for cellular energy production, metabolism, and redox signaling, mitochondria are inherently vulnerable to metal dyshomeostasis. Their double membrane is equipped with specific channels and transporters that regulate the flux of essential ions like Ca^2+^, K^+^, and transition metals such as Fe^2+^/^3+^ and Zn^2+^, making them a critical interface for metal-cell interactions [25]. Disruption of this delicate ionic balance is a fundamental driver of mitochondrial pathology. The mechanisms by which metals impair mitochondrial integrity are multifaceted, targeting core functions including bioenergetics, protein homeostasis, and quality control [26]. A key example is Cu. Excess Cu can induce mitochondrial protein misfolding, disrupting the function of the chaperone mitochondrial heat shock protein 70 (mtHsp70) and impairing biosynthesis. Upon entry into the matrix, Cu^2+^ inhibit essential enzymes like synthesis of cytochrome C oxidase 1 (SCO1), synthesis of cytochrome C oxidase 2 (SCO2), and cyclooxygenase (COX) subunits, which are crucial for the assembly and function of the electron transport chain (ETC), thereby crippling oxidative phosphorylation [27]. Similarly, Cd exposure compromises mitochondrial activity by directly inhibiting ETC complexes and precipitating the intrinsic apoptotic pathway, marked by mitochondrial membrane depolarization and cytochrome c release [3]. Other toxic metals, including Pb, Hg, and As, converge on similar pathways of ETC disruption and ROS overproduction.

This metal-induced damage ultimately overwhelms the mitochondrial quality control (MQC) system [28]. The failure of MQC, coupled with direct ETC impairment, leads to a pathological surge in ROS, creating a vicious cycle of oxidative stress and further mitochondrial decay. This cascade is particularly detrimental in post-mitotic, high-energy-demand cells. Consequently, neurons and cardiomyocytes exhibit heightened susceptibility, directly linking metal-associated mitochondrial dysfunction to the pathogenesis of major age-related diseases, including neurodegenerative and cardiovascular disorders [25,26,28]. Thus, the mitochondrion is not merely a passive target but a dynamic amplifier of metal toxicity, integrating specific ionic insults into a common pathway of cellular energy crisis and death. In addition to chemical inducers such as metal ions, mitochondria, organelles with a unique double-membrane structure, are also critical targets of ionizing radiation in radiotherapy. Radiation exposure triggers a cascade of mitochondrial alterations, including mitochondrial DNA (mtDNA) damage, ROS burst, and disruption of energy metabolism, which collectively influence cell fate decisions. The nature of radiation-induced mitochondrial dysfunction is closely associated with radiation quality, particularly the linear energy transfer (LET). High-LET radiation has been shown to predominantly induce point mutations in mtDNA, a key driver of mitochondrial impairment. Previous studies indicate that various types of mtDNA deletions can reversely activate DNA damage response (DDR) proteins through signaling pathways such as nuclear factor-kappa B (NF-κB)/phosphoinositide 3-kinase (PI3K)/protein Kinase B (Akt)/mechanistic target of rapamycin (mTOR) [17]. This bidirectional crosstalk between DDR and mitochondrial integrity provides a rational foundation for innovative combination strategies. Integrating radiation-induced mitochondrial genomic instability and metabolic dysregulation with metal ion-mediated mitochondrial stresses could establish a multi-target network that amplifies tumor cell death. Such an integrated approach holds promise for enhancing therapeutic efficacy and overcoming resistance in oncology.

### 2.3. DNA Damage

Metal-induced genomic instability represents a fundamental pathway through which metal ions execute their cytotoxic effects, directly threatening cellular integrity and viability. The assault on the genome occurs via dual, often synergistic, mechanisms: direct chemical interaction and indirect oxidative onslaught. Certain metal ions can directly bind to DNA, forming mutagenic adducts that distort the helix, cause base modifications, and induce strand breaks. More prevalently, the indirect pathway is mediated by metal-driven oxidative stress, where the generation of ROS launches a widespread attack on nucleic acids, leading to oxidation of bases and phosphodiester backbone breaks. To counteract this damage, cells activate the sophisticated DDR signaling network [29,30]. This surveillance system, coordinated by apical kinases like Ataxia Telangiectasia Mutated (ATM) and ATM and Rad3-related (ATR), orchestrates cell cycle arrest to allow for repair and, if damage is irreparable, initiates programmed cell death via pathways involving p53 [31,32]. Critically, metals can subvert this very defense system. They can directly inhibit the activity of core repair enzymes (e.g., DNA polymerases, ligases) and downregulate the expression of DDR proteins, thereby crippling repair capacity and fostering damage accumulation [33].

Cd serves as a prototypical example of this multifaceted genotoxicity. As a pervasive environmental carcinogen, Cd induces DNA damage both by triggering ROS-mediated oxidative damage and, insidiously, by impairing essential repair mechanisms like Base Excision Repair (BER) [33,34]. This dual action, inflicting damage while simultaneously disabling the repair toolkit, exemplifies how metals drive genomic instability. This paradigm extends beyond Cd; other ions including Zn, Cu, Fe, nickel (Ni), and aluminum (Al) also contribute to DNA damage through varied mechanisms [35], collectively highlighting the disruption of genomic integrity as a central and convergent mechanism in metal ion-induced cell death and carcinogenesis. The specific mechanisms of damage and repair associated with key metal ions are summarized in the table below (Table 2). Additionally, it is important to recognize that DNA damage represents a convergent mechanism not only for metal ions but also for radiotherapy, which directly induces DSBs [15]. A critical commonality between these two modalities lies in their ability to impair the DDR, thereby inhibiting repair processes and promoting cell death. Consequently, combining radiotherapy with metal-based agents that disrupt DDR signaling such as those targeting key repair enzymes or checkpoint proteins, can synergistically enhance tumor cell radiosensitivity [36]. This combinatorial approach, which exploits the dual inhibition of DNA repair pathways, represents a promising strategy to overcome radiotherapy resistance and improve therapeutic outcomes in oncology.

### 2.4. Epigenetic Modification

Emerging evidence positions epigenetic reprogramming as a critical and pervasive mechanism through which metal ions exert long-lasting effects on cell fate, including the regulation of cell death. Beyond directly damaging macromolecules, metals can hijack the epigenetic machinery, including encompassing DNA methylation, histone modifications, and non-coding RNA expression, to alter the transcriptional landscape of key survival and death pathways [5,43]. For instance, DNA methylation regulates autophagy and tumor progression by silencing genes like Beclin-1 (BECN1) and autophagy-related 4A cysteine peptidase (ATG4A), while histone modifications (e.g., acetylation, phosphorylation) and non-coding RNAs finely tune the expression of critical death regulators such as p53, BCL2 associated X (BAX), and Caspase-8 [43]. Metal ions act as potent disruptors of this epigenetic equilibrium. They can directly inhibit or alter the activity of writers and erasers of epigenetic marks, such as DNA methyltransferases (DNMTs), histone deacetylases (HDACs), and histone methyltransferases (HMTs) [5,44,45]. A hallmark of metal exposure, exemplified by As and Cd, is the induction of a paradoxical epigenetic state: global DNA hypomethylation concurrent with site-specific hypermethylation [45,46]. This aberrant pattern can silence tumor suppressor and DNA repair genes while promoting genomic instability, thereby facilitating malignant transformation and serving as a potential biomarker of early exposure.

Furthermore, metals extensively reshape the non-coding RNA regulome. By modulating the expression of microRNAs (miRNAs) and long non-coding RNAs (lncRNAs), they indirectly govern vast gene networks involved in apoptosis, autophagy, and oxidative stress responses [45,47,48]. For example, As exposure reprofiles specific miRNAs that target these pathways, and lncRNAs have been implicated in mediating heavy metal-induced apoptosis [47]. Critically, these epigenetic alterations induced by metals are not merely bystander effects but represent a programmable “epigenetic memory” that can persist and influence cellular phenotypes long after exposure ceases. This positions the epigenetic axis as a dynamic and integrative regulatory hub in metal toxicity, connecting environmental exposure to sustained dysregulation of cell death programs, with profound implications for understanding carcinogenesis and developing epigenetic-targeted therapies.

## 3. Different Cell Fates Induced by Metal Ions

The classification of cell death has evolved from purely morphological descriptions to a molecular and functional paradigm that encompasses distinct biochemical executioner mechanisms and, critically, divergent immunological outcomes. This modern framework is particularly relevant when examining the cytotoxicity of metal ions, which do not merely induce passive necrosis but can selectively activate specific, regulated cell death modalities, such as apoptosis, pyroptosis, ferroptosis, and cuproptosis. Each of these pathways is governed by a unique molecular signature and, importantly, communicates with the immune system in a defined manner through the release of specific DAMPs and cytokines. Consequently, metal ions act as versatile upstream triggers or modulators within this cell death network. Studying how different metals engage this network provides a powerful lens for understanding the fundamental crosstalk between metabolic perturbation, cell fate decisions, and immune activation.

### 3.1. Programmed Cell Death (PCD)

**Figure 2:**
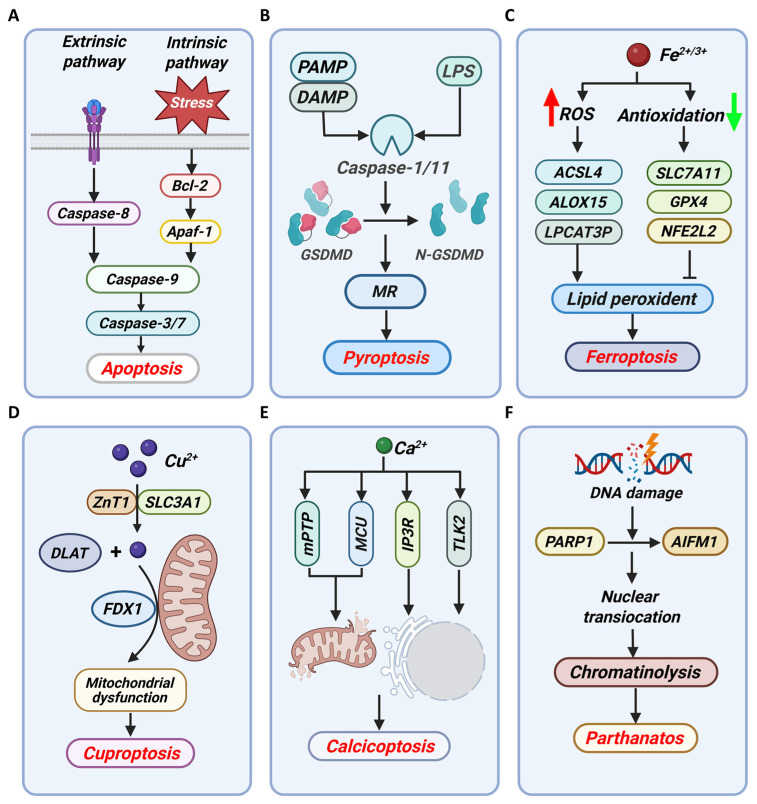
Schematic overview of PCD pathways. This figure illustrates the major molecular mechanisms underlying various forms of PCD triggered by metal ions and cellular stress signals. (**A**) Apoptosis: This pathway is divided into the extrinsic pathway, initiated by death receptor (e.g., Fas) ligation and leading to caspase-8 activation, and the intrinsic pathway, triggered by stress signals (e.g., ROS, DNA damage) that cause MOMP via BAX/BAK, cytochrome c release, and apoptosome formation to activate caspase-9. Both pathways converge on effector caspases-3/7 to execute cell death. (**B**) Pyroptosis: Triggered by PAMPs, DAMPs, or LPS, this pathway involves inflammasome (e.g., NLRP3) activation, leading to caspase-1/11 activation. These caspases cleave GSDMD into its N-GSDMD, which induces membrane rupture (MR), causing cell lysis and the release of inflammatory cytokines. (**C**) Ferroptosis: Driven by iron-dependent lipid peroxidation. Key regulators include system Xc^−^ composed of SLC7A11 and SLC3A1, which imports cysteine for GSH synthesis, and GPX4, which uses GSH to neutralize lipid peroxides. Enzymes like ACSL4, LPCAT3, and ALOX15 promote the formation and oxidation of PUFAs in membranes, while Nrf2 is a transcription factor that combats oxidative stress. (**D**) Cuproptosis: Induced by excessive Cu^2+^ ions, which are imported via transporters like ZnT1. Intracellular copper directly binds to lipoylated components of the TCA cycle, such as DLAT, causing their aggregation and the loss of Fe-S cluster proteins (e.g., via FDX1), leading to proteotoxic stress and cell death. (**E**) Calcicoptosis: Initiated by Ca^2+^ overload. Elevated cytosolic Ca^2+^ is taken up into mitochondria via the MCU, leading to mitochondrial Ca^2+^ overload. This triggers the opening of the mPTP, mitochondrial dysfunction, and the activation of calcium-dependent proteases, culminating in cell death. Ca^2+^ also causes the rupture of the endoplasmic reticulum membrane by IP3R and the rupture of the nuclear membrane by TLK2, ultimately leading to cell death. (**F**) Parthanatos: Triggered by severe DNA damage, this pathway leads to PARP1 hyperactivation, causing depletion of NAD^+^ and ATP. This bioenergetic crisis prompts the translocation of AIF from the mitochondria to the nucleus, where it induces large-scale DNA fragmentation and chromatinolysis. Created in BioRender. Shen, J. (2026) https://BioRender.com/d0g44u5.

Beyond causing passive necrosis, metal ions can serve as precise molecular switches that selectively activate distinct forms of PCD, including apoptosis, pyroptosis, necroptosis, and the recently characterized metal-dependent pathways of ferroptosis and cuproptosis (Figure 2). Each PCD modality is defined by a unique set of molecular initiators, effectors, and, most importantly, a characteristic “immunogenic fingerprint” conveyed through the release of specific DAMPs and cytokines. Therefore, the study of metal ion-induced PCD transcends mere toxicology; it places metal ions at the nexus of cellular metabolism, death signaling, and immune modulation.

#### 3.1.1. Apoptosis

Apoptosis, a quintessential form of PCD, is orchestrated through highly conserved intrinsic and extrinsic signaling pathways, culminating in hallmark morphological changes including cell shrinkage, chromatin condensation, and caspase-dependent dismantling of the cell. Beyond its fundamental role in development and homeostasis, apoptosis serves as a critical defense mechanism against malignant transformation, making its regulation a central focus in oncology. Metal ions have emerged as potent modulators of both apoptotic arms, acting not as mere toxins but as specific perturbators of its molecular rheostat. The extrinsic pathway is initiated by the ligation of death receptors (e.g., Fas) on the cell surface, leading to the assembly of the death-inducing signaling complex (DISC), activation of caspase-8, and subsequent execution via caspase-3 [49,50,51,52]. The intrinsic, or mitochondrial, pathway integrates diverse cellular stress signals, culminating in mitochondrial outer membrane permeabilization (MOMP) by BAX/BCL2 antagonist/killer 1 (BAK) proteins, release of cytochrome c, apoptosome formation with apoptotic protease-activating factor 1 (Apaf-1), and activation of caspase-9 [49,51,52]. These pathways converge on the activation of effector caspases that systematically execute the apoptosis process of cells (Figure 2A).

Critically, metal ions can engage this apoptotic machinery with precision. They can directly or indirectly promote ligand-independent clustering of death receptors or, more commonly, trigger the intrinsic pathway by inducing oxidative stress, mitochondrial dysfunction, and DNA damage. These core stressors can shift the balance of pro- and anti-apoptotic B-cell lymphoma 2 (BCL-2) family proteins towards MOMP. Currently, the therapeutic approaches that exploit this vulnerability have been exemplified by innovative nanomedicine strategies. For instance, Ma et al. designed a pH-responsive biomimetic nanoplatform, namely MGF@laN nanoparticles (MGF@laN NPs), that releases Ga^3+^ in the acidic TME to disrupt mitochondrial respiration and induce apoptosis, while co-released Mn^2+^ potentiates the cGAS-STING immune pathway via enhanced mitochondrial DNA sensing, promoting ICD [53]. This work underscores a sophisticated paradigm: metal ions can be engineered to co-opt apoptosis for a synergistic anti-tumor effect, combining direct cytotoxicity with immune activation. Despite these advances, a significant knowledge gap still persists. For example, while the intrinsic pathway is a well-established target for metal ions and nanomaterials, their specific and direct influence on the initiation and regulation of the extrinsic death receptor pathway remains underexplored. Thus, future research must systematically dissect how the physicochemical properties of metal-based nanomaterials (e.g., composition, size, surface charge) dictate their interaction with specific apoptotic components. Elucidating these interactions will be paramount for the rational design of next-generation metal-based therapies that can selectively modulate apoptotic thresholds in cancer cells.

#### 3.1.2. Pyroptosis

Pyroptosis represents a lytic and highly inflammatory form of PCD, serving as a critical bridge between innate immunity and cell death. Its execution is mediated by the gasdermin family of proteins, predominantly Gasdermin D (GSDMD). Upon cleavage by inflammatory caspases (e.g., caspase-1, caspase-4, caspase-5, caspase-11), the N-terminal fragment of GSDMD (N- GSDMD) oligomerizes to form plasma membrane pores, leading to cell osmotic swelling, rupture, and the massive release of pro-inflammatory cytokines (e.g., IL-1β and IL-18) [54,55,56] (Figure 2B). This mechanism positions pyroptosis not merely as a cell death pathway but as a potent alarm system that orchestrates immune surveillance. The canonical pyroptotic pathway is often initiated by cytosolic pattern recognition receptors that assemble into inflammasomes, with the NLRP3 inflammasome being the most extensively characterized sensor for diverse danger signals, including those derived from metal dyshomeostasis [10,56,57]. A key trigger for NLRP3 activation is potassium (K) ions efflux, a perturbation frequently induced by membrane-disrupting agents. This event promotes mitochondrial dysfunction and the generation of mitochondrial ROS (mtROS), facilitating the critical interaction between NIMA related kinase 7 (NEK7) and NLRP3, ultimately culminating in caspase-1 activation and GSDMD cleavage [10]. Thus, metal ions can instigate pyroptosis either directly by serving as NLRP3 agonists or indirectly by disrupting ionic gradients and mitochondrial integrity.

Harnessing this immunogenic potential for cancer therapy is a frontier in nanomedicine. Metal-based nanomaterials are particularly promising pyroptosis inducers due to their tunable physicochemical properties. For instance, Li et al. engineered a biodegradable NP (EI-NP) that inhibits calcium-dependent membrane repair, synergizing with a bacterial delivery system (VNP-GD) to potentiate GSDMD-mediated tumor pyroptosis [54]. Similarly, Engelke et al. developed lipid-coated Fe-based metal–organic framework NPs that release a burst of Fe^3+^ after lysosomal degradation. This release disrupts osmotic balance, directly inducing pyroptosis in tumor cells and triggering a concomitant anti-tumor immune response [58]. These strategies exemplify how metal ions can be structurally integrated into smart materials to achieve spatially controlled pyroptotic activation. The translational relevance of pyroptosis extends to combinatorial therapies. Notably, radiotherapy has been shown to activate the NLRP3/caspase-1/GSDMD axis, inducing pyroptosis that enhances the immunogenicity of irradiated tumor cells and recruits immune effector cells [57]. This creates a compelling rationale for combining radiotherapy with metal-based pyroptosis inducers or immunotherapies to amplify anti-tumor immunity. Therefore, targeting metal-induced pyroptosis transcends direct cytotoxicity, which represents a strategic modality to remodel the immunosuppressive TME and potentiate systemic immune responses, defining a new paradigm for metal-enabled oncotherapeutics.

#### 3.1.3. Ferroptosis

Ferroptosis has emerged as a paradigm-shifting form of PCD, distinguished by its unique metabolic basis at the intersection of Fe biology, redox homeostasis, and lipid peroxidation. It is mechanistically defined by the Fe-dependent accumulation of lethal lipid hydroperoxides, particularly on membranes rich in polyunsaturated fatty acids (PUFAs), a process driven by redox dysregulation [59,60]. Central to this pathway is the indispensable role of Fe, which acts as a dual-edged sword. First, labile Fe (e.g., Fe^2+^) fuels the Fenton reaction, generating highly reactive •OH that initiate lipid peroxidation. Second, Fe serves as an essential cofactor for enzymes like lipoxygenases (LOXs) that directly catalyze the oxidation of PUFAs, thereby propagating the peroxidation cascade [6,61]. The cellular defense against this ferroptotic cascade is primarily orchestrated by the GSH-GPX4 axis, where GPX4 utilizes GSH to reduce toxic lipid hydroperoxides to inert lipid alcohols [6,59,61]. Consequently, vulnerabilities in Fe metabolism (e.g., increased uptake) or the antioxidant defense (e.g., GPX4 inhibition) render cells exquisitely sensitive to ferroptosis (Figure 2C).

**Figure 3:**
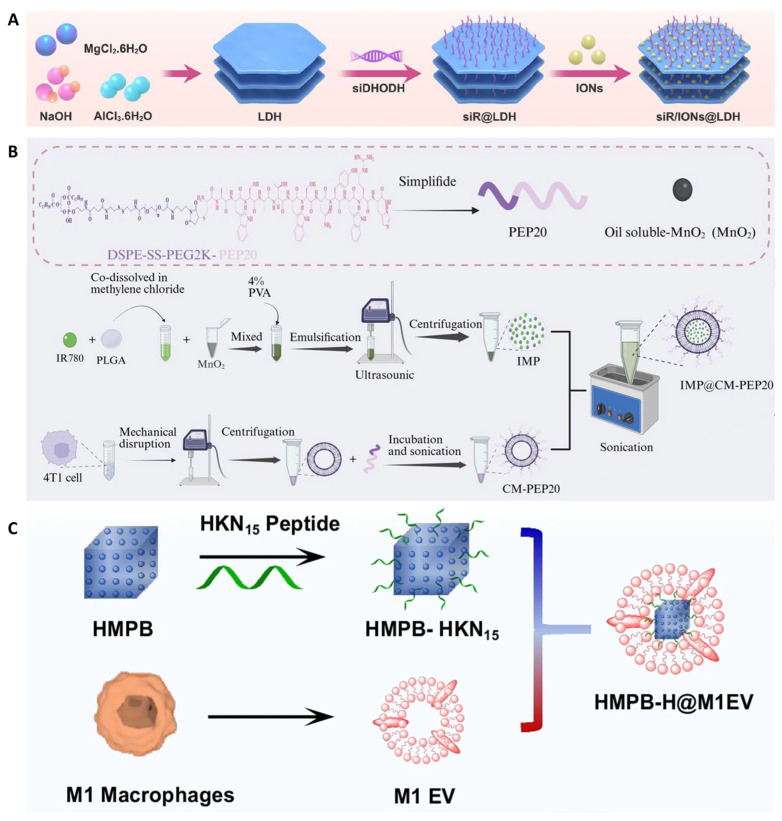
Multifunctional nanoplatforms for inducing ferroptosis in cancer therapy. (**A**) Schematic illustration of the construction and theranostic mechanism of the siR/IONs@LDH nanoplatform. Adapted with permission from Ref. [63]. Copyright 2023, ACS Applied Materials & Interfaces. (**B**) Schematic illustration of the IMP@CM-PEP20 NPs and their antitumor mechanisms. Adapted with permission from Ref. [64]. Copyright 2025, Journal of Nanobiotechnology. (**C**) Schematic illustration of ferritin-targeted biohybrids for immunotherapy, which activate endogenous iron and supply exogenous iron simultaneously to trigger ferroptosis. Adapted with permission from Ref. [65]. Copyright 2025, Nature Communications.

The induction of ferroptosis not only extends mere cytotoxicity, but represents a powerful metabolic intervention at the immuno-metabolic interface. Dysregulated Fe homeostasis in tumor cells profoundly influences both innate and adaptive immune responses, making ferroptosis a compelling target for immunotherapy [62]. This has catalyzed the rational design of sophisticated nanoplatforms that co-opt ferroptosis to ignite anti-tumor immunity. These platforms operate on three synergistic principles. The first principle focuses on amplifying the intrinsic Fenton reaction within tumors. For instance, Xu et al. engineered a layered double hydroxide nanoplatform (siR/IONs@LDH) that delivers a Fenton agent (ION) and a DHODH inhibitor (siR) to mitochondria. This combination disrupts the redox balance twice, by generating radicals and blocking detoxification, thereby dramatically sensitizing tumors to ferroptosis [63] (Figure 3A). Building on direct chemical potentiation, the second integrates metabolic intervention with immunologic checkpoint blockade. This approach is exemplified by the work of Liao et al. who developed a biomimetic nanoplatform (IMP@CM-PEP20) that concurrently induces ferroptosis and blocks the CD47 signal, promoting synergistic tumor clearance by combining metabolic killing with phagocytic immune activation [64] (Figure 3B). Ultimately, to maximize the ferroptotic drive, a third principle employs dual-source Fe amplification. Sheng et al. constructed a system (HMPB-H/M1EV) that both mobilizes endogenous Fe stores and delivers exogenous iron, creating a “dual-Fe” overload to maximally drive oxidative stress and ferroptosis, thereby triggering potent immune responses [65] (Figure 3C). Collectively, these strategies transcend the traditional view of metal ions as passive toxins. Instead, they exemplify a new therapeutic paradigm, the precise engineering of metal ion delivery and metabolism, to deliberately induce immunogenic ferroptosis. This approach actively reshapes the TME, turning a fundamental cell death mechanism into a tunable driver of systemic anti-tumor immunity, with vast potential for combination therapies.

#### 3.1.4. Cuproptosis

Cuproptosis has recently emerged as a paradigm-shifting, Cu-dependent form of PCD, distinguished by its unique mechanism centered on mitochondrial proteotoxic stress. Unlike canonical pathways (e.g., apoptosis, ferroptosis, pyroptosis), cuproptosis is initiated by the excessive accumulation of Cu^2+^ within mitochondria. Here, Cu^2+^ directly binds to lipid-acylated components of the tricarboxylic acid (TCA) cycle, prompting the aggregation of these acylated proteins and concurrent destabilization of iron–sulfur (Fe-S) cluster proteins [66,67,68,69] (Figure 2D). This dual proteotoxic crisis disrupts mitochondrial metabolism and energy production, ultimately triggering a distinct cell death signature [70]. The regulation of this pathway extends beyond Cu availability to involve intricate inter-metal competition. Recent work by Li et al. identified the Zn transporter ZnT1 as a critical mediator of Cu influx, essential for cuproptosis [71]. Structural analyses revealed that Zn^2+^ and Cu^2+^ compete for binding at the same site on ZnT1. Consequently, elevated Zn levels can inhibit Cu uptake via ZnT1, thereby suppressing cuproptosis. This discovery not only elucidates a fundamental regulatory node but also provides a mechanistic basis for using Zn supplementation in the clinical management of Wilson’s disease, a condition of pathological Cu overload [71].

**Figure 4:**
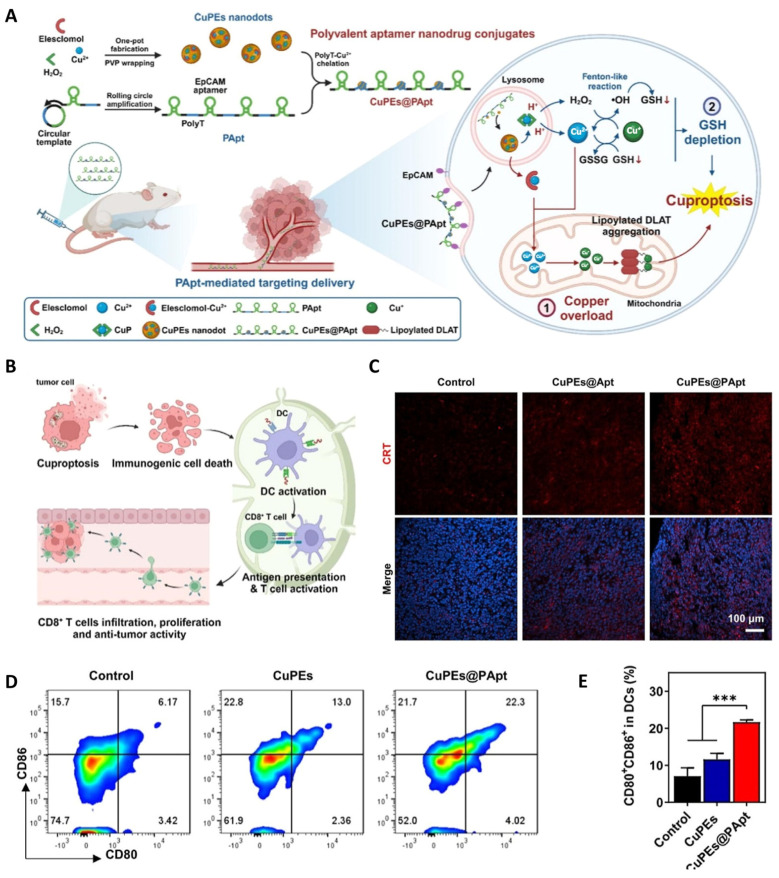
CuPEs@PApt nanoconjugates for cuproptosis induction and antitumor immune activation. (**A**) Schematic of the preparation of CuPEs@PApt and its “open-source cost-saving” mechanism to enhance tumor cuproptosis. (**B**) Schematic illustrating the antitumor immune activation process induced by CuPEs@PApt. (**C**) Representative IHC images of CRT staining in tumor tissues from mice treated with Control, CuPEs@Apt, or CuPEs@PApt. (**D**) Representative flow cytometry plots and (**E**) quantitative analysis of mature DCs (CD86^+^CD80^+^) in draining lymph nodes after intratumoral administration of the indicated treatments. *p* values were calculated by the Student’s *t*-test, *** *p* < 0.001. Adapted with permission from Ref. [73]. Copyright 2024, American Chemical Society.

In oncology, targeting cuproptosis presents a promising therapeutic strategy, particularly for malignancies exhibiting altered Cu metabolism. Current approaches bifurcate into two paradigms, either using Cu chelators to deplete oncogenic Cu pools, or employing Cu ionophores or carriers to deliberately induce cytotoxic Cu overload in cancer cells [67,72]. Nanotechnology is revolutionizing the latter strategy by enabling precise, targeted delivery. For instance, Wang et al. engineered a multivalent aptamer-based nanoconjugate (CuPEs@PApt) that combines mitochondrial Cu overload with glutathione depletion (Figure 4A). Upon internalization, these NPs trigger cuproptosis and subsequently induce ICD, as evidenced by increased calreticulin (CRT) exposure on the surface of tumor cells (Figure 4C). This ICD response promotes the maturation of dendritic cells (DCs) in draining lymph nodes, with flow cytometry showing elevated frequencies of CD86^+^CD80^+^ DCs (Figure 4D,E). The overall antitumor immune activation process is schematically depicted in Figure 4B [73]. Such rationally designed nanoplatforms exemplify how the fundamental biology of cuproptosis can be translated into sophisticated, multi-mechanistic therapeutics aimed at exploiting the inherent metabolic vulnerabilities of cancer cells.

Importantly, the immunogenic potential of cuproptosis-inducing therapies is increasingly recognized. The release of DAMPs from dying tumor cells, a hallmark of ICD, can orchestrate antitumor immune responses by promoting DC maturation and cytotoxic T lymphocyte infiltration (CTL) [18]. As demonstrated in the CuPEs@PApt system, the simultaneous induction of cuproptosis and oxidative stress not only amplifies direct tumor cell killing but also promotes CRT exposure and immune activation [73], highlighting the convergence of metal-induced cell death pathways with ICD-mediated immunotherapy.

#### 3.1.5. Calcicoptosis

Calcicoptosis represents a pivotal modality of cell death driven by the loss of intracellular Ca^2+^ homeostasis, positioning Ca^2+^ as a fundamental second messenger that can irrevocably switch from a signaling molecule to an executioner. This form of death is characterized by a self-amplifying cycle of cytoplasmic and mitochondrial Ca^2+^ overload, which engages multiple organelles and converges on irreversible mitochondrial failure. The initiating event is the pathological elevation of cytosolic Ca^2+^, which activates calcium-dependent phospholipases, compromising membrane integrity. Concurrently, this Ca^2+^ surge catalyzes a burst of ROS, instigating membrane lipid peroxidation and further disrupting cellular architecture [24,74]. It is mitochondria that act as the central executioner in this cascade. Cytosolic Ca^2+^ is rapidly taken up via the mitochondrial calcium uniporter (MCU), leading to matrix overload. This disrupts the electron transport chain, exacerbating ROS production. The resultant oxidative stress promotes the opening of the mitochondrial permeability transition pore (mPTP), collapsing the mitochondrial membrane potential and triggering a fatal positive feedback loop that seals cellular fate [24,75,76] (Figure 2E).

Additionally, the pathophysiological relevance of this pathway is starkly evident in neurodegenerative diseases, where excitotoxicity leads to sustained Ca^2+^ influx. In this context, Ca^2+^ activates proteases like calpain, which cleaves p35 to p25. Subsequently, the p25/Cdk5 complex phosphorylates the fission protein Drp1, promoting excessive mitochondrial fragmentation, energy crisis and neuronal death [77]. This delineates calcicoptosis as a critical mechanistic link between metabolic stress and neurotoxicity. In oncology, exploiting the inherent dysregulation of Ca^2+^ signaling in cancer cells has emerged as a promising therapeutic strategy. The goal is to achieve precise, tumor-specific Ca^2+^ overload using nanotechnology, thereby inducing lethal calcicoptosis while sparing normal tissue [78]. Currently, innovative nanoplatforms exemplify this approach. For instance, Zhang et al. developed hyaluronate-stabilized calcium peroxide NPs (SH-CaO_2_NPs) that decompose in the acidic TME, releasing Ca^2+^ and ROS directly within cancer cells [79]. Furthermore, more sophisticated systems aim to induce ICD. Pan et al. designed a PEGylated CaCO_3_-based nanomodulator (PEGCaCUR) that efficiently delivers Ca^2+^ to mitochondria, inducing overload while concurrently acting as an ICD inducer to potentiate anti-tumor immunity [80]. These strategies underscore a paradigm shift, moving beyond viewing calcium as a passive toxin toward engineering its delivery as a targeted, multi-mechanistic weapon that disrupts tumor metabolism and activates systemic immune surveillance.

#### 3.1.6. Parthanatos

Parthanatos has been defined as a unique, PARP1-dependent form of PCD programmed cell death that represents a critical convergence point for metabolic catastrophe and nuclear death. Its hallmark is the hyperactivation of PARP1 in response to severe genotoxic stress, leading to catastrophic depletion of nicotinamide adenine dinucleotide (NAD^+^) and adenosine triphosphate (ATP). This bioenergetic crisis precipitates the translocation of apoptosis-inducing factor (AIF) from the mitochondria to the nucleus, which orchestrates large-scale DNA fragmentation in a caspase-independent manner, distinguishing it from classical apoptosis and necrosis [81,82,83] (Figure 2F). Therefore, this pathway sits at the nexus of DDR, cellular metabolism, and epigenetic-like chromatin destruction.

Crucially, the oxidative stress and DNA damage induced by various metal ions serve as potent upstream triggers for this cascade. A variety of metals, including silicon (Si) [84], platinum (Pt) [85], and Zn [86], can initiate cell death by activating the Parthanatos pathway, which is strategically exploited in oncology. For instance, Zhou et al. demonstrated that specific palladium (II) complexes exert superior cytotoxicity against leukemia cells, including multi-drug resistant lines, predominantly by inducing Parthanatos, highlighting a rational design principle for novel metal-based chemotherapeutics [85]. Conversely, the role of metals can be protective in other contexts. Jiang et al. found that Zn promotes functional recovery after spinal cord injury by activating the SIRT3 pathway to mitigate oxidative stress and suppress mitochondrial dysfunction-associated Parthanatos [86]. This dual role underscores the context-dependent nature of metal-PARP1 interactions.

Beyond oncology, Parthanatos is increasingly implicated in the pathogenesis of major neurodegenerative diseases, including Parkinson’s, Alzheimer’s, and Huntington’s, where PARP1 overactivation contributes to neuronal loss [87]. Here, metal ion dyshomeostasis plays a part again. For example, Ca^2+^ overload can exacerbate the cycle of oxidative stress and energy failure that drives PARP1 activation. Targeting this intersection, Pan et al. identified the Ca^2+^ homeostasis regulator Calhm2 as a key mediator, whose inhibition effect alleviated α-synuclein-induced Ca^2+^ dysregulation and PARP1-dependent Parthanatos in Parkinson’s models, revealing a novel therapeutic node [88]. While PARP inhibitors are now cornerstone therapies in oncology, their translational potential for neurodegenerative diseases represents a promising but underexplored frontier, warranting dedicated clinical investigation. Thus, Parthanatos emerges not merely as a cell death subroutine, but as a dynamic mechanistic bridge connecting metal toxicity, metabolic integrity, and degenerative pathology across diverse disease states.

### 3.2. Non-Programmed Cell Death (Non-PCD)

Within the spectrum of cell death modalities, non-PCD represents a fundamental, passive form of cell death typically triggered by severe physicochemical insults or acute metabolic catastrophe. Unlike regulated pathways, it is not directed by dedicated molecular machinery but results from the abrupt collapse of cellular homeostasis, marked by rapid ATP depletion, loss of osmotic control, and irreversible membrane rupture. This process is strongly associated with acute injury and extreme stress, leading to the uncontrolled release of intracellular contents that potently initiate and amplify inflammatory responses. It is metal ions that are pivotal inducers of this lytic fate. By directly disrupting membrane integrity, depleting antioxidants to cause overwhelming oxidative damage, or inducing fatal ion dyshomeostasis, metals can force cells into this terminal, disorganized state. Thus, examining metal-induced non-PCD provides a critical lens beyond toxicology through which to understand acute tissue injury, sterile inflammation, and the subsequent immune microenvironment remodeling. The following sections will detail how specific metals drive this form of cell death and explore its implications in metal-associated diseases and potential therapeutic contexts.

#### 3.2.1. Necrosis

**Figure 5:**
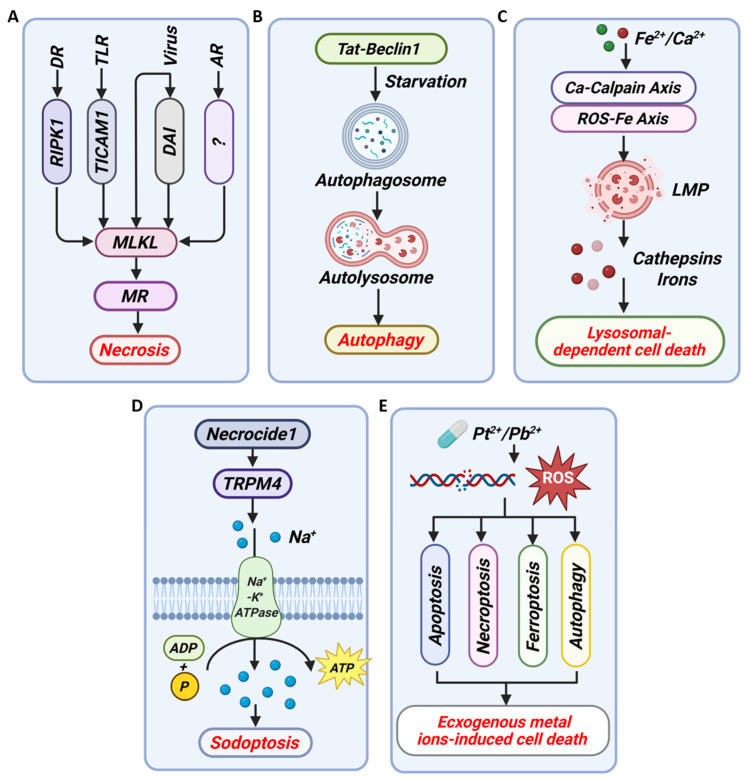
Schematic overview of non-programmed and exogenous metal ion-induced cell death pathways. This figure illustrates the major molecular mechanisms underlying various forms of non-PCD and cell death modalities triggered by exogenous metal ions, as discussed in Section 3.2 and Section 3.3. (**A**) Necrosis: This pathway can be initiated by DR, TLR, or viral infection via DAI. These signals lead to the activation of RIPK1 and RIPK3, which form the necrosome. The necrosome phosphorylates and activates MLKL, which oligomerizes to form pores in the plasma membrane, causing MR and lytic cell death. (**B**) Autophagy: Initiated by stressors like starvation or the Tat-Becin1 peptide, this pathway involves the formation of a double-membrane autophagosome that engulfs cellular material. The autophagosome fuses with a lysosome to form an autolysosome, where the contents are degraded. Dysregulated or excessive autophagic flux can lead to cell death. (**C**) Lysosomal-Dependent Cell Death: Triggered by Fe^2+^/Ca^2+^ overload, this pathway induces LMP via the ROS-Fe Axis and the Ca-Calpain Axis. LMP results in the leakage of cathepsins and other hydrolases into the cytosol, leading to cell death and crosstalk with other pathways like ferroptosis and autophagy. (**D**) Sodoptosis: Induced by molecules like Necrocide 1, which activates the TRPM4 channel. This leads to a massive influx of Na^+^, overwhelming the Na^+^/K^+^-ATPase pump. The resulting sodium overload causes osmotic imbalance, membrane depolarization, and rapid oncosis. (**E**) Pb and Pt-Induced Cell Death: Pb exposure induces oxidative stress by depleting antioxidants (e.g., SOD, GSH) and generating ROS. This damages mitochondria, leading to apoptosis. In contrast, Pt-based drugs (e.g., oxaliplatin) form DNA adducts, causing DNA damage that can trigger ICD, characterized by the release of DAMPs such as CRT and HMGB1. Created in BioRender. Shen, J. (2026) https://BioRender.com/az82az2.

Necrosis, once considered a passive and unregulated form of cell death resulting from extreme physicochemical insult, has been fundamentally redefined by the discovery of regulated necrotic pathways, most notably necroptosis. This paradigmatic shift positions necrosis not merely as a catastrophic endpoint but as a sophisticated, signal-dependent process integral to physiology, immunity, and disease. While characterized by hallmark features (e.g., plasma membrane permeabilization and the release of immunogenic cellular contents), regulated necrosis is executed by a precise molecular cascade. Its core machinery involves the activation of receptor-interacting protein kinases (e.g., RIPK1 and RIPK3), which form a dynamic signaling complex termed the necrosome. This complex ultimately phosphorylates and activates the mixed lineage kinase domain-like pseudokinase (MLKL), leading to its pore-forming oligomerization at the plasma membrane and lytic cell death [89,90] (Figure 5A). Critically, this pathway is intimately linked to metabolic and oxidative stress, as the necrosome can amplify mtROS production, creating a feed-forward loop that drives bioenergetic collapse [89].

Environmental and therapeutic metal ions are potent modulators of this pathway, with Ca, Pb, and As acting as potent inducers, often converging on oxidative stress as a central trigger. For instance, Ca^2+^ exposure upregulates the expression of RIPK1, RIPK3, and MLKL, directly engaging the core necroptotic machinery [19]. Similarly, Pb can induce necroptosis in neuronal models, associated with inflammatory cytokine production [19]. Beyond classic toxins, designed metal complexes are being explored for therapeutic necroptosis induction. Suntharalingam et al. demonstrated that rhenium (V) oxo complexes can directly induce necrosome-dependent ROS burst and mitochondrial dysfunction, showcasing the rational design of metallodrugs to activate this pathway [91]. Other metals (e.g., Zn and Cu) contribute to necrotic outcomes by causing severe membrane damage, ion dyshomeostasis, and overwhelming oxidative stress that bypasses specific regulation, leading to uncontrolled necrosis [69].

The immunogenic nature of necroptosis underpins its profound significance in disease pathophysiology and therapy. In oncology, necroptosis can overcome apoptosis resistance and expose tumor antigens, stimulating anti-tumor immunity [90]. Consequently, targeting the necroptosis pathway, particularly through MLKL modulation, has emerged as a promising strategy for cancers (e.g., colon carcinoma and melanoma). Conversely, dysregulated necroptosis is implicated in the pathogenesis of neurodegenerative and inflammatory diseases, where its inhibition may be therapeutic [89,90,91,92]. Thus, metal ions sit at a crucial interface, positioning them as both environmental triggers of pathological necrosis and potential tools for harnessing regulated necrosis for immunotherapy. This highlights a dual role that spans toxicology and translational medicine.

#### 3.2.2. Autophagic-Dependent Death

Autophagy, a highly conserved lysosomal degradation pathway, is crucial for maintaining cellular homeostasis by clearing damaged organelles, misfolded proteins, and intracellular pathogens in response to diverse stresses, including exposure to metal ions. Its execution is tightly regulated by signaling hubs like AMPK and mTOR and a core machinery of autophagy-related genes (ATGs) [93,94,95] (Figure 5B). Metal ions can critically perturb this delicate equilibrium, tipping the balance from cytoprotective autophagy towards a dysregulated, lethal autophagic flux.

Notably, the modulation of autophagy by metal ions is pleiotropic and dualistic. For instance, Ca^2+^ exerts bidirectional control via multiple pathways (e.g., CaMKKβ-AMPK-mTOR axis). Under basal conditions, Ca^2+^ signaling may suppress autophagy through mitochondrial communication, while elevated cytosolic Ca^2+^ can become a potent inducer of autophagic activity under stress [96,97]. In contrast, heavy metals like Cd^2+^ typically drive pathological autophagic dysregulation. Cd^2+^ exposure can upregulate key autophagic components (e.g., ATG7) yet simultaneously disrupt autophagic flux, inducing the complete process of autophagosome formation, fusion, and degradation. This disruption leads to the accumulation of dysfunctional autophagosomes and has been linked to enhanced proliferation, migration, and invasion in cancer cells (e.g., breast cancer), illustrating how metals can co-opt autophagy to promote oncogenic processes [94,98].

The link between dysregulated autophagy and disease pathogenesis underscores its therapeutic relevance. In oncology, deliberately metal ion-induced lethal autophagy in cancer cells has been an emerging strategy. Interestingly, the role of metals can be therapeutic in other contexts. For example, Cu^2+^ have been shown to alleviate lipid accumulation and oxidative stress in metabolic dysfunction-associated steatotic liver disease (MASLD) by restoring the chaperone-mediated autophagy of lipid droplets [99], which highlights the potential of precisely targeting the “metal ion-autophagy axis”. Therefore, elucidating how specific metals modulate distinct autophagic pathways extends beyond clarifying mechanisms of metal toxicity to uncover an innovative framework for developing novel metal-based anticancer therapies. The rational design of metallodrugs or nanomaterial, engineered to selectively trigger a lethal, dysregulated autophagic response in tumor cells, represents a promising translational frontier in leveraging this complex form of cell death for therapeutic gain.

#### 3.2.3. Lysosomal-Dependent Cell Death (LDCD)

LDCD represents a distinct, organelle-centric modality of cell death, positioning the lysosome, often termed the “suicide bag”, as an active executioner rather than a passive degradative endpoint. The pivotal event in LDCD is lysosomal membrane permeabilization (LMP), which results in the catastrophic release of potent hydrolases, particularly cathepsins, into the cytosol. This breach initiates a cascade of proteolytic degradation that dismantles key cellular structures and activates downstream death effectors [100,101,102]. The dual role of lysosomes in both homeostasis and death underscores a critical cellular checkpoint, whose stability dictates whether they serve as recycling centers or agents of destruction.

Metal ions are master regulators of this lysosomal checkpoint, primarily by inducing LMP through two convergent axes, namely the ROS-Fe Axis and the Ca-Calpain Axis. Lysosomes are exceptionally vulnerable to oxidative stress due to their high redox-active Fe content. Ions like Fe^2+^ can catalyze intra-lysosomal Fenton reactions, generating •OH that cause lipid peroxidation and direct membrane damage [103,104]. This Fe-mediated LMP is a key pathological mechanism, as evidenced by studies where quenching lysosomal Fe (e.g., with quercetin in alcoholic liver disease) mitigates cell death [103]. Concurrently, disruption of lysosomal Ca^2+^ homeostasis activates calcium-dependent proteases (e.g., calpain), which can directly destabilize the lysosomal membrane, creating a vicious cycle that amplifies LMP [105,106]. Other metal ions, including Cu^2+^, Al^3+^, Zn^2+^, and Mn^2+^, further contribute by catalyzing oxidative reactions or being released post-LMP to exacerbate cytosolic ROS bursts [100,101] (Figure 5C).

The profound role of LDCD in disease pathogenesis, especially in cancer and neurodegeneration, has catalyzed the development of lysosome-targeting metal-based therapeutics. The rationale is to exploit the inherent instability of cancer cell lysosomes, often termed “lysosomal cell death priming”. For instance, the agent BXQ-350 modulates lysosomal membranes to enhance anti-tumor activity [100]. More directly, engineered metal-based NPs, such as magnetic NPs (MNPs), can selectively accumulate in tumor lysosomes, inducing LMP, ROS overproduction, and synergistic activation of other death pathways like ferroptosis [101]. This multimodal mechanism helps overcome the frequent resistance of cancer cells to single-pathway therapies. Thus, the study of metal-induced LDCD transcends traditional toxicology, revealing the function of lysosomes as dynamic signaling hubs whose fate is governed by ionic balance. This understanding bridges fundamental cell biology with translational oncology, offering a paradigm where metals, both as environmental hazards and as rationally designed drugs, control a powerful cell death switch with profound implications for targeted cancer therapy.

#### 3.2.4. Sodoptosis

Sodium-induced cell death (or “sodoptosis”) represents an emerging paradigm of regulated necrosis, where the loss of sodium (Na) ions homeostasis acts as a direct biochemical executioner. Under physiological conditions, a steep Na^+^ gradient across the plasma membrane, maintained by the Na^+^/K^+^-ATPase, is critical for membrane potential, cellular volume, and secondary transport. The pathological intracellular accumulation of Na^+^, due to pump failure or channel dysregulation, leads to rapid plasma membrane depolarization, osmotic imbalance, catastrophic oncosis, and lytic death. This pathway is a key mediator of acute pathological stress in contexts such as neuroexcitotoxicity and ischemia–reperfusion injury, positioning Na^+^ not merely as an electrolyte but as a decisive factor in cell fate. Recently, the molecular machinery governing this form of death has been elucidated. A pivotal advance was the identification of Necrocide 1 (NC1), a small molecule that induces necroptosis through profound Na^+^ overload. NC1 directly targets and activates the transient receptor potential melastatin 4 (TRPM4) channel, a non-selective cation channel, triggering massive Na^+^ influx, immediate membrane depolarization, and rapid oncosis [107], which defined a specific regulatory modality of necrosis driven by sodium overload (NECSO) (Figure 5D). Notably, the therapeutic potential of manipulating this pathway is significant. For instance, in multiple myeloma, Sinobufagin promotes NECSO by stabilizing the TRPM4 protein, inhibiting its ubiquitin-proteasomal degradation and thereby exerting a potent anti-tumor effect [108].

Targeting the NECSO presents a promising, context-dependent strategy for cancer therapy. Dai et al. proposed a sophisticated combinatorial framework based on TRPM4 expression status, which stratifies tumors into two groups. For tumors with TRPM4 hypermethylation and low expression (e.g., colon adenocarcinoma), DNA methyltransferase inhibitors like decitabine could restore TRPM4 expression and re-sensitize cells to NECSO. Conversely, for tumors with high TRPM4 expression (e.g., endometrial cancer), combining TRPM4-mediated cell killing with targeted immune checkpoint inhibition (e.g., against PVRL2) could simultaneously induce direct tumor death and overcome immune evasion [109]. This approach highlights sodoptosis not as a simple cytotoxic endpoint, but as a programmable and immunologically relevant process that can be integrated into precision oncology paradigms. The exploration of metal ion-based strategies to selectively modulate this pathway, through direct channel activation or by disrupting ionic homeostasis, opens a novel frontier for developing therapies against cancers resistant to conventional apoptosis.

### 3.3. Exogenous Metal Ion-Induced Cell Death

Beyond the disruption of essential metal ion homeostasis, exogenous metal ions—particularly those administered as anticancer therapeutics—can directly induce cell death through distinct mechanisms. Two prominent examples are Pb and Pt, which, despite their contrasting roles as environmental toxicants and life-saving drugs, illustrate the dualistic nature of metal-induced cytotoxicity.

#### 3.3.1. Leadoptosis

Pb-induced cytotoxicity exemplifies a paradigm of metal toxicity, where the disruption of fundamental cellular processes converges to drive specific forms of cell death, aptly conceptualized within the framework of “leadoptosis”. Beyond its well-documented role as a pervasive environmental neurotoxicant, Pb exposure presents a critical model for understanding how a non-redox-active metal can orchestrate complex pathological cascades. Its insidious effects, particularly on the central nervous system, manifest as cognitive deficits and developmental delays, stemming from a synergistic assault on oxidative homeostasis, mitochondrial integrity, and neuroinflammatory signaling [110,111,112,113,114]. The core mechanism of leadoptosis is the subversion of redox balance. Pb indirectly amplifies oxidative stress by depleting endogenous antioxidants (e.g., GSH, SOD) and generating ROS, which in turn inflict macromolecular damage [115,116] (Figure 5E). This oxidative insult directly targets mitochondria, impairing their function and activating intrinsic apoptotic pathways, as demonstrated in neuronal models like HT-22 cells [114,117]. Furthermore, Pb perturbs key regulatory nodes like sirtuin 1 (SIRT1), leading to dysregulated autophagy, a process that can switch from protective to detrimental under sustained metal stress [117].

Notably, Pb toxicity is dynamically modulated through inter-metal interactions. For example, Pb can exacerbate Ca^2+^ signaling dysregulation, activating downstream stress kinases like ASK1 and p38 to promote neuronal death [118]. Conversely, protective trace elements like selenium (Se) can antagonize Pb toxicity by restoring antioxidant defenses and suppressing pro-inflammatory and pro-death pathways, particularly mitogen-activated protein kinase (MAPK)/NF-κB [119]. This crosstalk underscores that the cellular response to Pb is not isolated but integrated within a broader network. Current therapeutic strategies have been evolving from general detoxification to mechanism-targeted interventions. The focus is on counteracting oxidative stress and mitochondrial dysfunction, the primary drivers of leadoptosis. Biostimulants such as resveratrol [117] and melatonin [120] show promising potential in mitigating Pb-induced ROS and apoptosis. Additionally, molecules like ferritin that sequester labile Fe and inhibit Fenton reaction present a novel approach to disrupt the oxidative cascade at its source, thereby alleviating downstream cell death and mitophagy [121]. Elucidating the precise molecular signatures of leadoptosis opens avenues for developing targeted chelators or signaling modulators, moving the field towards precision interventions for metal-associated neurological and systemic disorders.

#### 3.3.2. Platinum-Induced Cell Death

In stark contrast to the accidental toxicity of Pb, Pt-based drugs, most notably cisplatin, carboplatin, and oxaliplatin [122], are deliberately administered as first-line chemotherapeutics for a wide range of malignancies [123]. While these agents share a common ability to form covalent DNA adducts that inhibit replication and transcription, their capacity to engage the immune system differs markedly. Oxaliplatin (OXA), in particular, is recognized as a prototypical type I inducer of ICD, a process characterized by the release of DAMPs such as CRT, ATP, and high-mobility group box 1 (HMGB1) [122,124]. These signals promote DC maturation, antigen presentation, and the priming of tumor-specific cytotoxic T lymphocytes (CTL), thereby converting the TME from immunosuppressive to immunostimulatory [123] (Figure 5E).

The therapeutic utility of Pt drugs is further amplified when combined with radiotherapy. Ionizing radiation induces DNA DSBs, which are predominantly repaired by NHEJ pathway. However, the presence of Pt-DNA adducts near DSB termini can severely compromise this repair process. As demonstrated by Boeckman et al., a single cisplatin 1,2-d(GpG) adduct located just seven base pairs from a DNA end reduces Ku binding by 50% and inhibits DNA-dependent protein kinase (DNA-PK) activity by up to 90%, effectively blocking end-joining in cell-free extracts [125]. This molecular blockade underlies the clinical observation that cisplatin must be administered before irradiation to achieve maximal synergy.

Recently, the integration of nanotechnology with Pt-based therapy has yielded sophisticated platforms designed to enhance ICD induction, overcome radioresistance, and amplify systemic anti-tumor immunity. For instance, Chen et al. developed gold (Au) nanocluster-mediated X-ray activatable Pt(IV) prodrugs, where photoelectron transfer under low-dose X-ray irradiation triggers the reduction of Pt(IV) to cytotoxic Pt(II) specifically within the tumor. This approach not only localizes DNA damage but also elicits robust ICD, sensitizing tumors to immune checkpoint blockade in breast cancer models [126]. A complementary strategy was reported by Wang et al., who constructed bimetallic OXA/Fe NPs. These particles respond to the acidic TME by releasing active OXA and ferrous ions, simultaneously inducing DNA damage and Fenton-like ROS generation. The resulting oxidative stress amplifies ICD post-radiotherapy and, importantly, triggers a strong abscopal effect, leading to regression of distant, non-irradiated tumors accompanied by long-term immunological memory [127]. In a distinct approach aimed at counteracting the immunosuppressive consequences of apoptosis, Zhang et al. engineered lipid NPs (LNPs) to co-deliver a cisplatin prodrug with Xkr8 siRNA and ANX5 mRNA. This formulation reduces the surface exposure of phosphatidylserine (PS) on dying tumor cells, thereby preventing the tolerogenic clearance by phagocytes and enhancing the immunogenicity of chemotherapy [128].

Other groups have explored novel Pt complexes that go beyond classical DNA damage. Tang et al. synthesized Pt(II)-NHC complexes derived from 4,5-diarylimidazoles, which not only induce DNA lesions and ROS production but also trigger endoplasmic reticulum stress, leading to upregulation of PD-L1 on tumor cells. This conversion of immunologically “cold” tumors into “hot” ones positions these complexes as promising partners for checkpoint inhibitor therapy in hepatocellular carcinoma [124]. Several studies have further demonstrated that Pt NPs can serve as versatile platforms for multi-agent delivery. Li et al. developed a “nanobraker” consisting of Pt cores coated with polymer-catechol, enabling co-delivery of the mTOR inhibitor TAK228 and an anti-PD-L1 antibody. The Pt core itself sensitizes tumors to TAK228-enhanced radiotherapy, while the released antibody blocks PD-1/PD-L1 signaling and downregulates oncogenic MYC and HIF-1α, crippling melanoma tumorigenesis and angiogenesis [129]. Collectively, these recent innovations illustrate how Pt-based nanoformulations can be engineered to achieve spatiotemporally controlled drug release, amplify ICD, activate innate immune pathways, and remodel the TME for enhanced checkpoint blockade immunotherapy. The integration of radiotherapy with these metal-based strategies not only overcomes local resistance but also establishes systemic antitumor immunity, a goal long sought in oncology.

Collectively, the study of exogenous metal ions, whether environmental toxicants (e.g., Pb) or therapeutic agents (e.g., Pt, Au), reveals a rich diversity of cell death mechanisms with profound implications for human health. While Pb serves as a model for metal-induced neurodegeneration, Pt drugs exemplify how metal-DNA interactions can be harnessed to trigger immunogenic cell death and synergize with radiotherapy and immunotherapy. Recent advances in nanotechnology have further expanded the therapeutic repertoire, enabling precise spatiotemporal control, robust activation of innate and adaptive immunity, and the conversion of localized treatments into systemic antitumor responses. Elucidating the precise molecular signatures of these metal-induced death pathways will continue to guide the development of next-generation metal-based therapeutics.

## 4. Application of Metal Ions in Cancer Therapies

The strategic deployment of metal ions represents a transformative frontier in oncology, evolving from the empirical use of classical chemotherapeutics (e.g., Pt agents) to the rational design of next-generation metallodrugs and nanoplatforms with targeted and immunomodulatory functions. This progression underscores a paradigm shift where metals are no longer viewed merely as cytotoxic payloads but as versatile central players that can be engineered to disrupt specific tumor vulnerabilities, modulate the immune microenvironment, and overcome therapeutic resistance. The convergence of bioinorganic chemistry, nanotechnology, and immunology has unlocked unprecedented opportunities to harness the unique redox, catalytic, and coordination properties of metal ions for precision cancer therapy. The following section will critically examine this expanding landscape. Finally, biological mechanisms and therapeutic applications of these metal ions will be systematically summarized in Table 3. This synthesis aims to not only chart the current state of the field but also to illuminate strategic pathways for translating metal-based immunotherapies into clinical practice, offering novel avenues for more effective and integrated cancer treatment.

### 4.1. Metal-Based Vaccine Adjuvants

Metal-based vaccine adjuvants represent a paradigm shift in immunoengineering, transitioning vaccine development from empirical formulations to the rational design of agents that actively direct immune responses. Adjuvants, enhancing antigen immunogenicity, function primarily by activating antigen-presenting cells (APCs) to bridge innate and adaptive immunity [12,139]. While traditional Al salts (e.g., alum) remain a cornerstone for inducing potent antibody responses, their limited capacity to stimulate robust cell-mediated immunity, a critical requirement for anti-tumor vaccines, has driven the search for novel adjuvants capable of qualitatively shaping the immune response [12,139].

**Figure 6:**
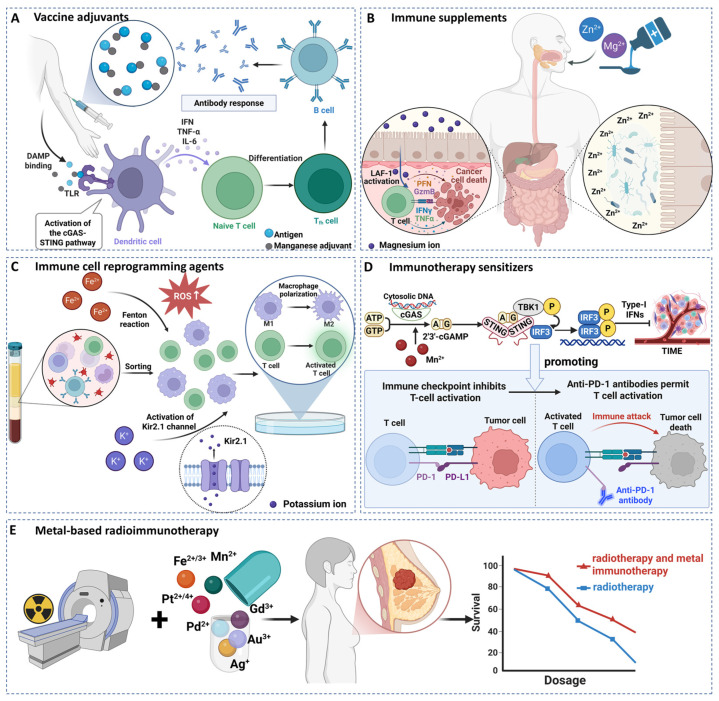
Metalloimmunotherapies. (**A**) Vaccine adjuvants. For example, Mn^2+^ can be used as an adjuvant to enhance the anti-tumor immune response of vaccines. Created in BioRender. Shen, J. (2026) https://BioRender.com/kjxuuff. (**B**) Immune supplements. For example, Mg^2+^ can maintain the activity of LAT-1 and kill tumors; Zn^2+^ can inhibit pathogenic bacteria and maintain the intestinal physical barrier. Created in BioRender. Shen, J. (2026) https://BioRender.com/5k5j1t0. (**C**) Immune cell reprogramming agents. For example, Fe^2+^ and K^+^ can reprogram T cell and macrophages to enhance anti-tumor capacity. Created in BioRender. Shen, J. (2026) https://BioRender.com/bq15ccg. (**D**) Immunotherapy sensitizers. For example, Mn^2+^ combined with immune checkpoint inhibitors can reverse immunosuppression. Created in BioRender. Shen, J. (2026) https://BioRender.com/cywyhhi. (**E**) Metal-based radioimmunotherapy. For example, Fe^2+/3+^ and Pt^2+/4+^ can enhance the efficacy of radiotherapy and promote the treatment of triple negative breast cancer. Created in BioRender. Shen, J. (2026) https://BioRender.com/392w3i4.

Within this pursuit, specific metal ions have emerged as powerful and tunable innate immune stimulants. Notably, manganese ion (Mn^2+^) has been identified as an endogenous “second messenger” for the cGAS-STING pathway. It potently lowers the activation threshold of cGAS for cytosolic DNA, promoting the production of type I interferons and pro-inflammatory cytokines even in the absence of infection, thereby positioning Mn^2+^ itself as a potent adjuvant [9,139,140] (Figure 6A). Leveraging this, Luo et al. developed a spleen-targeted mRNA vaccine (Mn@mRNA-LNP) that co-delivers Mn^2+^ with tumor antigen-encoded mRNA. This platform drives DC maturation, cross-presentation, and a potent antigen-specific CD8^+^ T cell response [132]. Additionally, Zn^2+^ plays a distinct yet complementary role as an “allosteric modulator” of the same pathway, facilitating the phase separation of cGAS upon DNA binding to license its enzymatic activity [9]. Exploiting this synergy, Sun et al. engineered Zn-Mn-CDN coordination NPs (ZMCP), as a self-adjuvanting delivery system, eliciting strong anti-tumor immunity and serving as an effective carrier for protein antigens like those from severe acute respiratory syndrome coronavirus 2 (SARS-CoV-2) [141].

Concurrently, nanomaterial engineering provides a critical vector to overcome the limitations of classical metal salts and precisely control adjuvant activity. For instance, porous Al-based metal–organic framework NPs (Al-MOF NPs) have been designed as inhalable lung adjuvants, addressing the poor pulmonary tolerance of traditional alum and opening avenues for mucosal vaccination [131]. These engineered platforms allow for the spatiotemporal control of metal ion release, targeted delivery to specific immune cells, and the coordinated presentation of antigens and immunostimulatory signals. Taken together, the field is evolving from using metals as simple “immunoenhancing particles” to employing them as “programmable immune signal conductors”. The convergence of bioinorganic chemistry, nanotechnology, and systems immunology positions metal-based adjuvants as a cornerstone for the next generation of therapeutic cancer vaccines and broadly protective antiviral strategies.

### 4.2. Metal-Based Immune Supplements

Beyond their direct therapeutic applications, essential metal ions function as fundamental immunonutritional supports, strengthening systemic immune competence by maintaining metabolic homeostasis and cellular integrity. Deficiencies in these micronutrients broadly undermine both innate and adaptive immune functions, highlighting their indispensable role in sustaining the body’s foundational defense capacity rather than acting as direct immunostimulants [142]. Among numerous metal ions, magnesium ion (Mg^2+^) exemplifies this supportive role. As a vital cofactor in hundreds of enzymatic reactions, including those central to cellular energy metabolism (e.g., ATP synthesis), the availability of Mg^2+^ is crucial for the basic bioenergetics of all immune cells [143]. Recent mechanistic insights, as the described “Mg^2+^-lymphocyte function-associated antigen 1 (LFA-1) axis”, elucidate how Mg^2+^ sufficiency optimizes the adhesion and migration efficiency of CD8^+^ T cells, thereby enhancing their functional readiness (Figure 6B). Conversely, dietary Mg^2+^ deficiency correlates with impaired infection control and weakened tumor surveillance, underscoring its role in maintaining immune fitness [133]. Thus, Mg^2+^ supplementation can be viewed as restoring optimal physiological conditions for immune cell performance.

Zn^2+^ operates as a cornerstone of systemic and mucosal immunity, primarily through its role in maintaining barrier integrity and cellular proliferation. Adequate Zn^2+^ plays a critical role in the normal development and function of the immune system, and its deficiency leads to thymic atrophy and reduced lymphocyte counts [144,145]. A key aspect of its immune-supportive function is its regulation of the gut-immune axis. Zn^2+^ is essential for preserving intestinal epithelial barrier function via the upregulation of tight junction proteins (e.g., zonula occludens-1 [ZO-1]) and supportive signaling pathways (e.g., PI3K/AKT/mTOR) [146,147]. It also helps maintain a balanced gut microbiota, and deficiency leads to dysbiosis and inflammation [148,149] (Figure 6B). Studies confirm that Zn^2+^ deficiency disrupts the cellular composition and immune function of secreting secretory immunoglobulin A (sIgA) in gut-associated lymphoid tissue (GALT), which can be reversed upon Zn^2+^ repletion [134]. This underscores the fundamental role of Zn^2+^ in supporting the body’s first line of defense and overall immune homeostasis. Therefore, Zn- and Mg-based nutritional supplementation strategies aim primarily to correct deficiencies and reinstate optimal physiological homeostasis. This approach strengthens the host’s overall constitution and resilience, potentially improving outcomes in conditions from infections to cancer as an adjunctive measure [144].

### 4.3. Metal Ion-Based Immune Cell Reprogramming Agents

The metabolic dysregulation within the TME represents a fundamental mechanism of immunosuppression, in which cancer cells co-opt nutrient resources and establish a metabolically hostile niche that cripples effector immune cell function. This concept extends beyond the classical Warburg effect, which is the preferential use of glycolysis by tumors, to encompass its immunological consequence. These consequences occur specifically as resulting lactate accumulation and nutrient depletion directly impair the metabolic fitness, proliferation, and cytotoxic activity of effector T cells (Teffs), while paradoxically supporting the differentiation and function of immunosuppressive regulatory T cells (Tregs) [11,150]. To reverse this state of functional paralysis, immune cell metabolic reprogramming has emerged as a strategic frontier. This approach aims not merely to inhibit tumors but to therapeutically rewire the intracellular metabolism of immune cells, thereby restoring or enhancing their anti-tumor capabilities. This reprogramming targets two pivotal immune populations: T cells and macrophages. Initially, naïve T cells are in a metabolically quiescent state. Their successful activation and differentiation into Teffs following antigen encounter depend on a rapid shift to aerobic glycolysis and oxidative phosphorylation [11,151]. However, the TME actively suppresses this essential metabolic transition. Similarly, macrophages undergo dynamic polarization from pro-inflammatory, anti-tumor M1 phenotypes to pro-tumorigenic, immunosuppressive M2 phenotypes, a shift driven by metabolic cues from the TME [11,150,152].

Metal ions serve as powerful metabolic modulators and signaling co-factors in this reprogramming process. For macrophages, Fe plays a crucial role. Nanocomposites based on Fe oxide can enhance antigen cross-presentation and promote the repolarization of TAMs towards the M1 phenotype [13] (Figure 6C). Furthermore, platforms that leverage the Fenton reaction driven by Fe^2+^ can deplete intratumoral glucose while generating cytotoxic ROS, creating a dual metabolic stress that forces M2-to-M1 repolarization [13]. Beyond Fe, targeting ion channels disrupted in the TME, such as the Kir2.1 potassium channel that inhibits TAM function, represents another promising avenue for functional restoration [136] (Figure 6C). For T cell reprogramming, the challenge lies in overcoming exhaustion and poor infiltration. Innovative “bidirectional nanomodulators” have been designed to address this. For instance, a pH-responsive nanosystem co-delivering Mn^2+^ and siPD-L1 executes a dual strategy whereby Mn^2+^ acts as a STING pathway agonist to promote DC activation, Teff infiltration, and ICD, while siPD-L1 concurrently knocks down the immune checkpoint PD-L1 on tumor cells to reactivate exhausted T cells [135]. This approach exemplifies how metals can be engineered to simultaneously manipulate tumor cell signaling and immune cell metabolism. Metal ion-based immune cell reprogramming transcends direct cytotoxicity. It represents a sophisticated form of metabolic immunotherapy that seeks to correct the immunosuppressive metabolome of the TME and empower the host’s immune system. Future progress hinges on developing agents with greater cellular specificity, understanding the long-term metabolic imprint of reprogramming, and strategically combining these approaches with checkpoint inhibitors to achieve synergistic and durable anti-tumor immunity.

### 4.4. Metal Ion-Based Immunotherapy Sensitizers

The therapeutic efficacy of immune checkpoint inhibitors (ICIs) is fundamentally constrained by the immunosuppressive TME, which fosters primary and acquired resistance. This “cold” TME is characterized by dominant immunosuppressive networks, including Tregs, myeloid-derived suppressor cells (MDSCs), and a milieu of inhibitory cytokines, that actively impede T cell infiltration and function, rendering ICIs ineffective [153]. To overcome this barrier, metal ion-based immunotherapy sensitizers have emerged as a strategic class of agents. Their primary mode of action is not to directly kill tumor cells or reprogram immune cell metabolism, but to pharmacologically remodel the TME, thereby reversing immunosuppression and converting immunologically “cold” tumors into ICI-responsive “hot” tumors. These sensitizers function by targeting key immunosuppressive nodes within the TME. A prominent strategy involves activating the innate immune sensing machinery, particularly the cGAS-STING pathway, which is a critical mediator for generating an immunogenic TME and this pathway for which Mn^2+^ serves as potent endogenous agonists [154,155]. When delivered via nanoplatforms (e.g., MnO_2_ nanosheets), Mn^2+^ potently activates the cGAS-STING axis in tumor and antigen-presenting cells. This triggers a cascade of type I interferon and pro-inflammatory cytokine release, which in turn reprograms the cellular composition of the TME. It promotes the infiltration and activation of cytotoxic CD8^+^ T cells and M1 macrophages while concurrently counteracting the immunosuppressive landscape that blunts ICI activity [138] (Figure 6D). Thus, Mn^2+^ acts as a microenvironmental “primer”, establishing a conducive context for checkpoint blockade.

Another approach targets adaptive response to stress of the tumor. Cobalt ions (Co^2+^) can mimic hypoxia and stabilize hypoxia-inducible factor-1α (HIF-1α), a master regulator involved in tumor angiogenesis and immune evasion. By modulating the HIF-1 pathway, Co^2+^ compounds can theoretically alter vascular permeability and immune cell trafficking within the TME, potentially disrupting the physical and biochemical barriers that limit ICI efficacy [137]. However, the role of Co^2+^ is complex and context-dependent, as chronic HIF-1α stabilization may also promote pro-tumorigenic pathways, underscoring the need for precise spatiotemporal control in its therapeutic application. To conclude, metal ion-based sensitizers represent a paradigm of combinatorial immunotherapy focused on TME. By selectively disrupting the immunosuppressive networks that shield tumors, through pathways like cGAS-STING or hypoxia signaling, they functionally convert an immunosuppressive TME into an immunologically active one. This creates a permissive environment where ICIs can effectively engage the pre-existing but suppressed anti-tumor immune response. The future of this field lies in engineering targeted delivery systems that maximize TME-specific activity while minimizing systemic effects, thereby safely unlocking the full potential of ICIs across a broader spectrum of malignancies.

### 4.5. The Synergistic Effect of Radiotherapy and Metal Immunotherapy

Radiotherapy, a cornerstone of local tumor treatment, exerts its therapeutic efficacy largely through the immune response it elicits. Substantial evidence confirms that radiotherapy can activate the immune system by inducing ICD. The ROS generated during irradiation disrupt nuclear integrity, leading to the release of HMGB1. Simultaneously, increased ROS levels within the endoplasmic reticulum promote the exposure of CRT. These ICD-associated DAMPs facilitate DC activation and migration, thereby priming T cells and eliciting a systemic anti-tumor immune response. However, the immunosuppressive TME frequently results in inadequate immune activation or acquired resistance following radiotherapy. Metal ions and their nanomaterial counterparts offer a versatile platform to overcome this limitation. Numerous high-atomic-number metal NPs function as effective radiosensitizers by enhancing localized energy deposition. Their favorable chemical stability, low toxicity, and structural diversity make them particularly suitable for biomedical applications. Extensive research has been conducted on NPs composed of Au, Pt, bismuth (Bi), iridium (Ir), gallium (Ga), and tellurium (Te), aiming to augment the radiosensitivity of malignant cells [156] (Figure 6E).

Beyond physical radiosensitization, rationally designed metal-based NPs can simultaneously address tumor hypoxia, a major barrier to effective radiotherapy. For instance, Yang et al. developed PEGylated Au-Pt NPs that not only enhance DNA damage via physical dose deposition but also exhibit catalase-like activity, catalyzing the decomposition of endogenous H_2_O_2_ to O_2_ within the TME, thereby alleviating hypoxia and overcoming hypoxia-induced radioresistance [157]. This dual-action strategy exemplifies the potential of engineering metal-based nanoplatforms to combine physical and biochemical radiosensitization.

**Figure 7:**
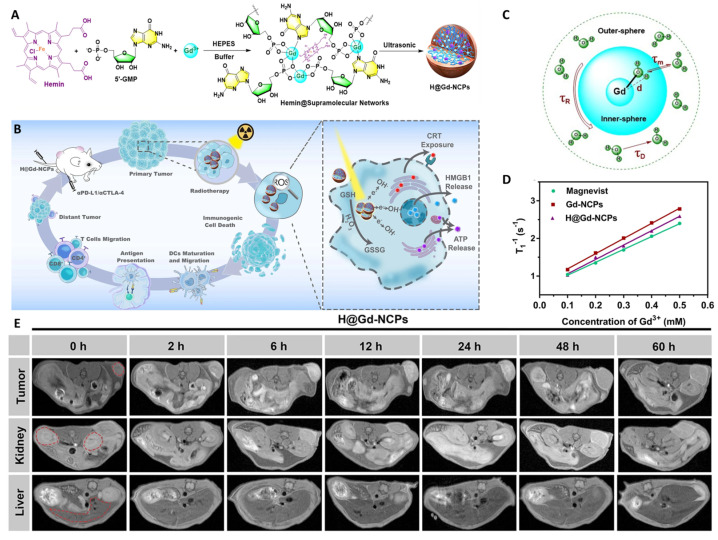
H@Gd-NCPs as a radiosensitizer and immunopotentiator. (**A**) Schematic illustration of the preparation of H@Gd-NCPs. (**B**) Mechanism by which H@Gd-NCPs achieve radiosensitization through amplifying intracellular oxidative stress and potentiating checkpoint blockade immunotherapy. (**C**) Schematic diagram of the MRI principle. (**D**) Longitudinal relaxivity (r_1_) values of Magnevist, Gd-NCPs, and H@Gd-NCPs (*n* = 3 biologically independent samples; experiment repeated twice independently with similar results). (**E**) Dynamic MRI after intravenous injection of H@Gd-NCPs ([Gd^3+^] = 30 mg kg^−1^) in vivo, and the dashed red circles indicated tumor, kidney and liver, respectively (*n* = 3 biologically independent animals). Adapted with permission from Ref. [158]. Copyright 2021, Nature Communications.

Consequently, combining radiotherapy with metal-based agents has emerged as a promising strategy to amplify oxidative stress induced by radiotherapy, thereby potentiating ICD and robust anti-tumor immunity. As evidenced by recent studies, Huang et al. developed a novel radiosensitizer based on a hemin@Gd^3+^/5′-GMP nano-coordination polymer [158]. As shown in Figure 7A, H@Gd-NCPs were synthesized via supramolecular self-assembly of hemin, Gd^3+^, and 5′-GMP. The mechanism of radiosensitization involves dual amplification of oxidative stress: Gd-mediated enhancement of X-ray energy deposition increases •OH production, while hemin catalyzes GSH depletion, thereby potentiating radiation-induced ICD and sensitizing tumors to checkpoint blockade immunotherapy (Figure 7B). Furthermore, H@Gd-NCPs serve as effective T_1_-weighted MRI contrast agents, with longitudinal relaxivity (r_1_) values surpassing those of the commercial agent Magnevist (Figure 7C,D). In vivo MRI imaging further demonstrated significant tumor accumulation of H@Gd-NCPs, with enhanced signal intensity persisting for up to 24 h post-injection (Figure 7E). This multifunctional nanoplatform exemplifies how integrating high-Z elements with redox-modulating components can amplify radiotherapy-induced immunogenic cell death and improve the efficacy of radioimmunotherapy.

To address insufficient immune activation by radiotherapy in triple-negative breast cancer (TNBC), Wang et al. constructed an oxygen-self-supplying nano-radiosensitizer comprising FePt alloy and MnO nanocrystals. This system activates both ICD and the cGAS-STING pathway, strengthening the anti-tumor immune response and improving radioimmunotherapy outcomes. Through catalytic cascade reactions mediated by FePt, it generates ROS and O_2_ within tumor cells, thereby attenuating tumor hypoxia and enhancing radiotherapeutic efficacy. The damage mediated by ROS robustly induces ICD in TNBC, promotes DC maturation and CTL infiltration, and ultimately synergizes with cancer immunotherapy [159]. Moreover, the combination of radiotherapy and metal ions can remodel the post-radiation immune microenvironment and reverse radiotherapy resistance. For instance, a Cu-based nano-coordination polymer developed by Wang et al. independently and simultaneously induces Cu-dependent •OH production and GSH consumption, achieving potent ICD induction in synergy with radiotherapy. Importantly, this combinatory approach significantly enhances DC maturation and intratumoral infiltration of anti-tumor CD8^+^ T cells, thereby potentiating immune checkpoint blockade therapy against both primary and metastatic tumors [160]. Collectively, the integration of radiotherapy with metal immunotherapy not only provides physical and chemical radiotherapy sensitization but also enables multipronged immunomodulation. This synergistic strategy offers a promising avenue to overcome current limitations in oncology by concurrently enhancing local tumor control and systemic antitumor immunity.

## 5. Conclusions and Prospects

This review systematically delineates the diverse modalities of cell fate induced by metal ions, including apoptosis, pyroptosis, ferroptosis, cuproptosis, and necrosis, unifying them under a central paradigm, which holds that metal ions act as fundamental molecular switches that govern cellular fate through mechanisms such as oxidative stress, mitochondrial dysfunction, DNA damage, and epigenetic reprogramming. Beyond cytotoxicity, these ions are critical immune regulators, dynamically shaping T cell function, antigen presentation, inflammatory responses, and the TME, thereby establishing the conceptual and therapeutic foundation for the emerging field of metal immunotherapy. This convergence of cell death mechanisms and immunomodulation represents the core academic contribution of this work.

Despite rapid progress, the translation of metal immunotherapy faces several interrelated challenges. The metabolic interdependence and crosstalk among metal ions complicate precise physiological targeting [161]. Moreover, their biological effects are highly context-dependent, varying across cell types and microenvironments, thus necessitating the development of selective delivery systems for cell and tissue. Finally, the overlapping molecular networks governing different metal-induced death pathways require more rigorous mechanistic definition and standardized detection methodologies [161]. To advance the field, future research should prioritize several key directions. Efforts should focus on the development of high-resolution, dynamic imaging tools for real-time tracking of metal ion distribution and speciation in vivo [162], and concurrently, the rational design of intelligent, responsive nanomaterials that enable spatiotemporally controlled release and action of metal ions at disease sites [163]. Additionally, exploring the combination of traditional therapies (e.g., radiotherapy) with metal immunotherapy will be crucial to firmly establish metal-based agents as viable immunotherapeutics [161]. Collectively, metal ions, among the most ancient biological signaling molecules, are now recognized as powerful and tunable mediators of cell death and immunity. A deeper understanding of their precise mechanisms not only elucidates fundamental biological principles but also provides innovative paradigms for treating cancer and other diseases. With continued interdisciplinary integration and technological innovation, metal immunotherapy is poised to become a transformative pillar of precision medicine.

## Figures and Tables

**Table 1:** Characteristics of oxidative stress induced by some metal ions.

Metal Ions	The Main Sources of ROS	Specific Injury Marker	Type of Inducing Cell Death	Ref.
Fe^2+^	Fenton reaction	Lipid peroxidation	Ferroptosis	[23,24]
			Apoptosis	
			Necroptosis	
			Autophagy	
Cu^2+^	Mitochondrial electron transport chain	Protein aggregation	Cuproptosis	[23,24]
			Autophagy	
Zn^2+^	NADPH oxidase	Lysosomal membrane permeabilization	Lysosomal-dependent cell death	[24]
			Autophagy	
Ca^2+^	Mitochondrial electron transport chain Endoplasmic reticulum stress	Lipid	Calcicoptosis	[24]
		Protein	Apoptosis	
		Nucleic acid peroxidation	Autophagy	
Cd^2+^	NADPH oxidase	DNA damage	Ferroptosis	[19,23]
			Autophagy	
			Apoptosis	
			Necroptosis	
Pb^2+^	Mitochondrial superoxide	Neurotoxicity	Ferroptosis	[19]
			Apoptosis	
			Necroptosis	
As^3+^	Nitric oxide synthase	Chromosomal aberration	Ferroptosis	[19,23]

**Table 2:** The mechanisms of DNA damage induced by metal ions.

Types of DNA Damage	Major Induced Metal	Characteristic Repair Pathway	Metal Specific Effects	Ref.
Oxidative base damage	Fe	BER	Inhibition of OGG1 and MTH1	[37]
	Cu			
Strand break	Cr	HR NHEJ	Inhibition of PARP1 and XRCC1	[35,38,39,40]
	As			
	Cd			
Intra-chain/inter-chain cross-links	Pt	NER	Inhibition of ERCC1-XPF	[35,41,42]
	Cr			

Abbreviations: BER, base excision repair; HR, homologous recombination; NHEJ, non-homologous end joining; NER, nucleotide excision repair; OGG1, 8-oxoguanine DNA glycosylase; MTH1, MutT homolog 1; PARP1, poly (ADP-ribose) polymerase 1; XRCC1, X-ray repair cross complementing 1; ERCC1-XPF, ERCC1-XPF nuclease; ERCC1, ERCC excision repair 1, endonuclease non-catalytic subunit; XPF, ERCC excision repair 4 (ERCC4), endonuclease catalytic subunit.

**Table 3:** Strategies for metal ions in tumor immunotherapy.

Strategies	Representative Metal	Main Mechanism	Advantages of Treatment	Clinical Progress	Reference
Vaccine adjuvants	Mn	Activating cGAS-STING Enhancing APC function	Improving the vaccine titer Inducing long-lasting immunity	Preclinical Early clinical	[9,130,131,132]
	Zn				
	Ca				
	Al				
Immune supplements	Mg	Optimizing the function of immune cells Improving metabolism	Reducing immune senescence Enhancing treatment tolerance	Multiple clinical studies	[133,134]
	Zn				
Immune cell reprogramming agents	K	Regulating cell metabolism and differentiation Enhancing effector functions	Improving the efficacy of adoptive cell therapy	Preclinical Studies	[13,135,136]
	Mn				
	Fe				
Immune sensitizers	Mn	Activating innate immunity Reversing immunosuppression	Expanding the population benefiting from immunotherapy	Preclinical Early clinical trials	[137,138]
	Co				

## Data Availability

No primary research results, software or code have been included and no new data were generated or analyzed as part of this review.

## Abbreviations

The following abbreviations are used in this manuscript:

Cd	cadmium
Pb	lead
Hg	mercury
As	arsenic
Fe	iron
Cu	copper
Zn	zinc
Ni	nickel
Al	aluminum
K	potassium
S	sulfur
Si	silicon
Pt	platinum
Na	sodium
Se	selenium
Mn	manganese
Mg	magnesium
Co	Cobalt
Au	gold
Bi	bismuth
Ir	iridium
Ga	gallium
Te	tellurium
Gd	gadolinium
DAMP	damage-associated molecular pattern
cGAS	cyclic GMP-AMP synthase
STING	stimulator of interferon genes
NLRP3	NOD-like receptor thermal protein domain-associated protein 3
TAM	tumor-associated macrophage
TME	tumor microenvironment
DSB	DNA double-strand break
ROS	reactive oxygen species
mtROS	mitochondrial ROS
ICD	immunogenic cell death
GSH	glutathione
Nrf2	nuclear factor erythroid 2-related factor 2
Hmox1	heme oxygenase 1
Gsta3	glutathione S-transferase alpha 3
SOD	superoxide dismutase
GPX4	glutathione peroxidase 4
•OH	hydroxyl radical
ETC	electron transport chain
mtHsp70	mitochondrial heat shock protein 70
SCO1	synthesis of cytochrome C oxidase 1
SCO2	synthesis of cytochrome C oxidase 2
MQC	mitochondrial quality control
COX	cyclooxygenase
mtDNA	mitochondrial DNA
DDR	DNA damage response
NF-κB	nuclear factor-kappa B
PI3K	phosphoinositide 3-kinase
Akt	protein Kinase B
mTOR	mechanistic target of rapamycin
BER	base excision repair
HR	homologous recombination
NHEJ	non-homologous end joining
NER	nucleotide excision repair
OGG1	8-oxoguanine DNA glyco-sylase
MTH1	MutT homolog 1
PARP	poly(ADP-ribose) polymerase 1
XRCC1	X-ray repair cross complementing 1
ERCC1-XPF	ERCC1-XPF nuclease
ERCC1	ERCC excision repair 1, en-donuclease non-catalytic subunit
XPR	ERCC excision repair 4 (ERCC4), endonuclease catalytic subunit
BECN1	Beclin-1
ATG4A	autophagy-related 4A cysteine peptidase
DNMT	DNA methyltransferase
HDAC	histone deacetylase
HMT	histone methyltransferase
miRNA	microRNA
lncRNA	long non-coding RNA
BAX	BCL2 associated X
BAK	BCL2 antagonist/killer 1
PCD	programmed cell death
Apaf-1	apoptotic protease-activating factor 1
NEK7	NIMA related kinase 7
LOX	lipoxygenase
PUFA	polyunsaturated fatty acid
TCA	tricarboxylic acid
MCU	mitochondrial calcium uniporter
mPTP	mitochondrial permeability transition pore
NAD^+^	nicotinamide adenine dinucleotid
ATP	adenosine triphosphate
PARP1	poly ADP-ribose polymerase 1
AIF	apoptosis-inducing factor
RIPK	receptor-interacting protein kinase
MLKL	mixed lineage kinase domain like pseudokinase
ATG	autophagy-related gene
MASLD	metabolic dysfunction-associated steatotic liver disease
LDCD	lysosome-dependent cell death
LMP	lysosomal membrane permeabilization
NC1	Necrocide 1
TRPM4	transient receptor potential melastatin 4
NECSO	necrosis driven by sodium overload
SIRT1	sirtuin 1
MAPK	mitogen-activated protein kinase
APC	antigen-presenting cell
DC	dendritic cell
SARS-CoV-2	severe acute respiratory syndrome coronavirus 2
GALT	gut-associated lymphoid tissue
Teff	effector T cell
Treg	immunosuppressive regulatory T cell
ZO-1	zonula occludens-1
ICI	immune checkpoint inhibitors
MDSC	myeloid-derived suppressor cell
sIgA	secretory immunoglobulin A
HIF-1α	hypoxia-inducible factor-1α
HMGB1	high-mobility group protein B1
CRT	calreticulin
TNBC	triple-negative breast cancer
DNA-PK	DNA-dependent protein kinase
CTL	cytotoxic T lymphocytes
OXA	Oxaliplatin
PS	phosphatidylserine
LPS	lipopolysaccharide
ACSL4	acyl-CoA synthetase long-chain family member 4
SLC7A11	solute carrier family 7 member 11
ALOX15	arachidonate 15-lipoxygenase
LPCAT3	lysophosphatidylcholine acyltransferase 3
NFE2L2	nuclear factor erythroid 2-related factor 2
GSDMD	gasdermin D
N-GSDMD	N-terminal fragment of GSDMD
BCL-2	B-cell lymphoma 2
SLC3A1	solute carrier family 3 member 1
DLAT	dihydrolipoamide S-acetyltransferase
FDX1	ferredoxin 1
IP3R	inositol trisphosphate receptor
AIFM1	apoptosis-inducing factor mitochondria associated 1
MR	membrane rupture
AR	androgen receptor
DR	death receptor
TLR	Toll-like receptor
TICAM1	TIR domain-containing adaptor molecule 1
DAI	DNA-dependent activator of IFN-regulatory factors
ATM	Ataxia Telangiectasia Mutated
ATR	ATM and Rad3-related
